# Potent, p53-independent induction of NOXA sensitizes *MLL*-rearranged B-cell acute lymphoblastic leukemia cells to venetoclax

**DOI:** 10.1038/s41388-022-02196-y

**Published:** 2022-01-28

**Authors:** Klaudyna Fidyt, Agata Pastorczak, Julia Cyran, Nicholas T. Crump, Agnieszka Goral, Joanna Madzio, Angelika Muchowicz, Martyna Poprzeczko, Krzysztof Domka, Lukasz Komorowski, Magdalena Winiarska, Joe R. Harman, Karolina Siudakowska, Agnieszka Graczyk-Jarzynka, Elzbieta Patkowska, Ewa Lech-Maranda, Wojciech Mlynarski, Jakub Golab, Thomas A. Milne, Malgorzata Firczuk

**Affiliations:** 1 Department of Immunology, Medical University of Warsaw, Warsaw, Poland; 2 Postgraduate School of Molecular Medicine, Medical University of Warsaw, Warsaw, Poland; 3 Department of Pediatrics, Oncology and Hematology, Medical University of Lodz, Lodz, Poland; 4 Medical Research Council Molecular Haematology Unit at the University of Oxford, Oxford, UK; 5 Mossakowski Medical Research Institute, Polish Academy of Sciences, Warsaw, Poland; 6 Department of Hematology, Institute of Hematology and Transfusion Medicine, Warsaw, Poland

**Keywords:** Sequencing, Acute lymphocytic leukaemia, Paediatric cancer

## Abstract

The prognosis for B-cell precursor acute lymphoblastic leukemia patients with *Mixed-Lineage Leukemia* (*MLL*) gene rearrangements (MLLr BCP-ALL) is still extremely poor. Inhibition of anti-apoptotic protein BCL-2 with venetoclax emerged as a promising strategy for this subtype of BCP-ALL, however, lack of sufficient responses in preclinical models and the possibility of developing resistance exclude using venetoclax as monotherapy. Herein, we aimed to uncover potential mechanisms responsible for limited venetoclax activity in MLLr BCP-ALL and to identify drugs that could be used in combination therapy. Using RNA-seq, we observed that long-term exposure to venetoclax in vivo in a patient-derived xenograft model leads to downregulation of several tumor protein 53 (*TP53*)-related genes. Interestingly, auranofin, a thioredoxin reductase inhibitor, sensitized MLLr BCP-ALL to venetoclax in various in vitro and in vivo models, independently of the p53 pathway functionality. Synergistic activity of these drugs resulted from auranofin-mediated upregulation of NOXA pro-apoptotic protein and potent induction of apoptotic cell death. More specifically, we observed that auranofin orchestrates upregulation of the NOXA-encoding gene Phorbol-12-Myristate-13-Acetate-Induced Protein 1 (*PMAIP1*) associated with chromatin remodeling and increased transcriptional accessibility. Altogether, these results present an efficacious drug combination that could be considered for the treatment of MLLr BCP-ALL patients, including those with *TP53* mutations.

## Introduction

B-cell precursor acute lymphoblastic leukemia (BCP-ALL) is a genetically heterogeneous disease caused by clonal proliferation of immature B cells, which occurs both in children and adults. The WHO classification of BCP-ALL based on specific chromosomal rearrangements and ploidy identifies several genetic subtypes, which greatly differ in therapeutic responses. Rearrangements of the *Mixed-Lineage Leukemia* (*MLL*) gene, which encodes lysine methyltransferase 2A (KMT2A), constitute one of the high-risk (HR) subtypes and predict dismal survival prognosis. Among many different *MLL* translocation partners, the *AF4* gene is most frequently found in BCP-ALL [1]. A hallmark of MLL fusion protein activity is the presence of increased histone H3 lysine 79 dimethylation (H3K79me2) at gene targets due to recruitment of DOT1L [2–4]. Besides, MLL fusion proteins also recruit a large molecular weight complex containing many other factors involved in promoting transcription elongation [5–9]. MLLr BCP-ALL is characterized by a low level of somatic mutations and a very poor response to therapy, rendering this subtype essentially incurable. Novel effective combination treatment strategies for the MLLr BCP-ALL are urgently needed.

One of the prominent features of cancer cells conferring resistance to chemotherapy is the upregulation of anti-apoptotic B-cell lymphoma-2 (BCL-2) family proteins. The BCL-2 family controls the initiation of intrinsic apoptosis, the major cell death pathway triggered in response to chemotherapeutics. It comprises pro-apoptotic (e.g., BAX, BAK, BIM, BID, NOXA) and anti-apoptotic (e.g., BCL-2, MCL-1, BCL-XL) members [10, 11]. Among the BCL-2 family, BCL-2 itself is most comprehensively studied and linked to chemotherapy failure [12, 13]. In 2015, venetoclax (VEN), the first-in-class selective BCL-2 inhibitor, was approved for the treatment of chronic lymphocytic leukemia (CLL) patients who failed previous treatment [14, 15]. The antitumor activity of VEN has also been demonstrated in preclinical studies in other hematological cancers including BCP-ALL [16]. Initially, the efficacy of VEN was shown for two HR BCP-ALL subtypes, *TCF3-HLF*-positive [17] and MLLr BCP-ALL [18]. Subsequently, it was demonstrated that VEN exerts antileukemic effects also in other BCP-ALL subtypes, such as Philadelphia chromosome-positive (Ph-positive) [19] and hypodiploid [20].

Despite the strong rationale for targeting BCL-2 in HR BCP-ALL, the response of leukemic cells to VEN is heterogeneous [19, 20] and VEN monotherapy is frequently insufficient to completely eliminate leukemic cells [18, 21–23]. Thus, rather than being used as a monotherapy, the efficacy of VEN is currently being tested in clinical trials in combination with conventional chemotherapy for the treatment of relapsed/refractory (R/R) BCP-ALL, including patients with MLLr BCP-ALL (ClinicalTrials.gov: NCT03808610, and NCT03504644). Nevertheless, the broad spectrum of action and severe side effects caused by chemotherapy make identification of other drugs that could sensitize HR BCP-ALL cells to VEN an important issue.

In this work, to better select a drug for the combination treatment with VEN, we investigated alterations in gene expression in MLLr BCP-ALL cells subjected to VEN in vivo. In VEN-treated cells, we found significant dysregulation of the p53 pathway, which may have suppressed apoptotic signaling. Therefore, we investigated whether auranofin (AUR), a drug that can induce apoptosis independently of p53 [24], could synergize with VEN. AUR is a thioredoxin reductase (TXNRD) inhibitor that is approved for the treatment of rheumatoid arthritis. Importantly, enzymes of the thioredoxin (TXN) system protect cells from apoptosis [25]. AUR triggers mitochondrial stress [26], modulates levels of pro-apoptotic and anti-apoptotic proteins, and demonstrates antitumor activity [27]. Previously, we have shown that AUR effectively kills BCP-ALL cells of various subtypes including MLLr BCP-ALL [28]. Here we found that AUR potentiates VEN activity against MLLr BCP-ALL cells in vitro and in vivo. We explored the mechanism of the synergistic interaction and found that it is mediated by the pro-apoptotic protein NOXA induced by AUR in a p53-independent manner.

## Results

### Treatment with VEN attenuates the p53 pathway in MLLr BCP-ALL primary cells

**Fig. 1:**
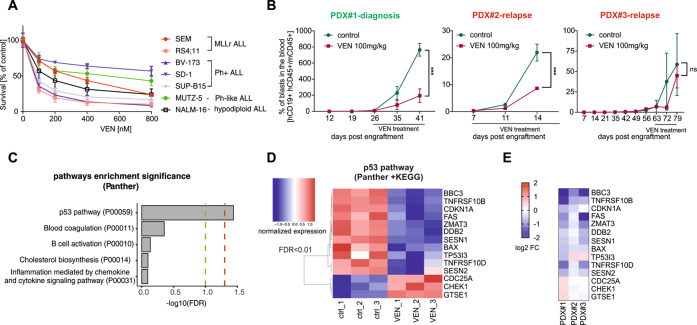
Long-term in vivo VEN treatment affects p53 pathway in MLLr BCP-ALL PDXs. **A** BCP-ALL cell lines were treated with VEN for 48 h and subjected to MTT viability assay. The viable cells are presented as a percentage of DMSO-treated cells (control). The bars represent mean ± SD from two independent experiments for each cell line. **B** MLLr BCP-ALL PDXs (PDX#1, PDX#2 and PDX#3; adult marked in green, pediatric marked in red color) were injected into NSG mice and the leukemic blast engraftment was measured by murine peripheral blood staining. Once the engraftment was confirmed [human cells (hCD45^+^, hCD19^+^) were detected in blood among murine leukocytes (mCD45^+^)], mice received VEN at a dose of 100 mg/kg through oral gavage (o.g.), and control mice received the solvent (60% Phosal 50PG; 30% PEG400; 10% ethanol) in a total of 13 doses (in cycles 5 days treatment/2 days off; between 26 and 42 days post engraftment) (PDX#1), daily for 11 days (between 7 and 17 days post engraftment) (PDX#2) or in 12 doses (in cycles 5 days treatment/2 days off; between 63 and 78 days post engraftment) (PDX#3). The graph shows the progression of leukemia over time as presented by the mean percentage of human blasts among murine mCD45^+^ cells ± SD (*n* = 3 per group). Statistical significance of the interaction effect was estimated by a two-way ANOVA test, ***P < 0.001. **C** p53 pathway as most significantly enriched pathway among differentially expressed genes (DEGs) from RNA-seq performed in PDX#1 treated with vehicle or VEN in vivo as described in (**B**). Dashed lines are indicated for FDR = 0.05 (red) and FDR = 0.1 (green). **D** Gene expression heatmap of the DEGs from RNA-seq performed on PDX#1 cells following long-term VEN exposure in vivo, as described in (**B**). The graph shows top DEGs from p53 pathway with FDR < 0.01. Each column represents an individual sample. **E** Heatmap presents the mRNA levels of the genes from the p53 pathway shown in (**D**) in three MLLr PDXs following long-term in vivo exposure to VEN, relative to untreated controls (log2FC). The mRNA levels were assessed by RT-qPCR, using gene-specific TaqMan probes. The mean expression levels in VEN-treated and control groups were calculated from independent measurements for 2 (PDX#1) or 3 (PDX#2, PDX#3) mice.

To compare the cytotoxicity of VEN against MLLr and other BCP-ALL subtypes we employed a panel of BCP-ALL cell lines including MLLr (SEM, RS4;11), Ph-positive (BV-173, SD-1, SUP-B15), Philadelphia-like (Ph-like) (MUTZ-5) and hypodiploid (NALM-16). As presented in Fig. 1A, VEN exerted heterogeneous efficacy, even within the same subtype, with EC50 values ranging from 40 to 800 nM. In primary and primograft cells (PDXs) derived from pediatric and adult BCP-ALL patients, VEN was cytotoxic in most cases, with an EC50 range of 4.8 × 10^−3^ to 1.8 μM (Supplementary Fig.1), which is achievable after oral administration of VEN (*C*_max_ = 1–3 μM) [14]. In contrast, we observed variable in vivo efficacy of VEN against MLLr BCP-ALL PDXs. Specifically, in three out of six tested PDXs, VEN halted leukemia progression (Supplementary Fig. 2A), but in the other three PDXs we detected increasing number of leukemic cells in murine blood despite the treatment (Fig. 1B), indicating that in some MLLr PDXs VEN monotherapy is insufficient to completely halt leukemia progression. Therefore, to determine pathways that could account for the limited efficacy of VEN, we investigated global changes in gene expression upon prolonged in vivo exposure to VEN. Following the last dose of VEN, leukemic cells were isolated from the spleens of control and VEN-treated mice, by enriching for human CD19^+^ cells. The cells isolated from PDX#1 were subjected to RNA sequencing (RNA-seq). Analysis of differentially expressed genes (Supplementary Fig. 2B, C) revealed significant dysregulation of the p53 pathway-associated genes in VEN-treated group (Fig. 1C, D). Specifically, we observed pronounced downregulation of several groups of p53 target genes including key pro-apoptotic BCL-2 family members (*BAX*, *BBC3*), cell cycle inhibitors (*CDKN1A*, *ZMAT3*), and receptors involved in pro-apoptotic signaling (*FAS*, *TNFRSF10A*, *TNFRSF10B*). Simultaneously, we detected increased expression of genes inhibited or downregulated by p53 (*CDC25A*, *CHEK1*), as well as the gene which attenuates p53 transcriptional activity (*GTSE1*) (Fig. 1D). In agreement with the RNA-seq data (from PDX#1), we found a downregulation of the majority of the p53 target genes in all three MLLr PDXs as assessed by RT-qPCR (Fig. 1E and Supplementary Fig. 2D). Altogether, these results suggest impairment of p53-dependent cell cycle arrest and apoptosis in MLLr BCP-ALL cells upon in vivo treatment with VEN.

### AUR enhances the response of BCP-ALL cells to VEN in vitro and in vivo

Limited in vivo VEN efficacy and attenuation of the p53 pathway suggested that drugs working independently of p53 might be better candidates for combination therapy with VEN. Hence, we selected AUR, an inhibitor of the TXN system that demonstrated activity against MLLr BCP-ALL PDXs in our previous studies [28] and has been shown to work independently of p53 in lymphoma models [24]. Importantly, AUR treatment killed the MLLr BCP-ALL cell lines RS4;11 and SEM with similar efficacy (Supplementary Fig. 3A), despite differences in their *TP53* status. RS4;11 harbors wild-type *TP53* [29], whereas SEM contains R288Q pathogenic variant (Supplementary Fig. 3B). Consistent with the increased stability of this p53 variant, we detected the p53 protein in SEM cells by immunoblotting, unlike in other BCP-ALL cell lines containing WT p53 (Supplementary Fig. 3C,D) [30].

**Fig. 2:**
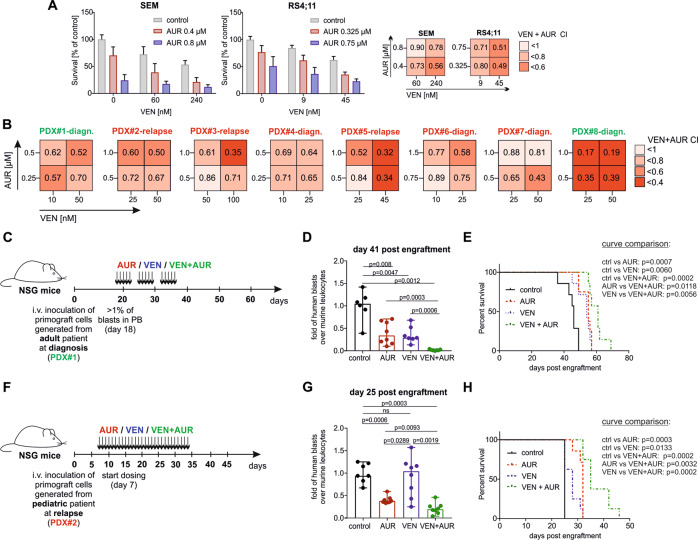
AUR augments VEN activity against MLLr BCP-ALL cell lines and PDXs in vitro and in vivo. **A** SEM and RS4;11 MLLr BCP-ALL cell lines were treated with EC20 and EC50 concentration of VEN or AUR (calculated by nonlinear regression dose-response analysis using GraphPad7), as well as with a combination of both drugs for 48 h. Subsequently, cells were subjected to MTT assay. The viable cells are presented as the percentage of untreated control (DMSO). Bars represent mean + SD, *n* = 8. Based on the drug response, the combination index (CI) was calculated using CompuSyn software employing Chou-Talalay algorithm [57]. Right panel shows CI matrices representing particular VEN+AUR CI values. CI = 1 shows the additive effect, CI < 1 indicates the synergistic effect of the drugs. **B** CI matrices presenting antileukemic activity of VEN+AUR tested in MLLr BCP-ALL PDXs (*n* = 8). Pediatric (marked in red color) and adult (marked in green color) MLLr PDXs derived at diagnosis (*n* = 5) and relapse (*n* = 3) were co-cultured with primary BM-MSC cells and treated with VEN, AUR or a combination of both for 4–5 days. After treatment, the total amount of viable cells was determined by flow cytometry using anti-CD19 antibody, 7AAD dye and counting beads. CI value for each drug combination was calculated with CompuSyn. **C** In vivo experimental scheme. PDX#1 cells were injected into NSG mice and disease progression was determined weekly based on peripheral blood staining. When the percentage of human leukemic cells (hCD45^+^, hCD19^+^) reached 1% of murine CD45^+^ cells (day 18), VEN and AUR administration was initiated. Randomly selected mice were treated through o.g. with 100 mg/kg of VEN, intraperitoneally (i.p.) with 10 mg/kg of AUR, a combination of both drugs or control (DMSO – i.p., 60% Phosal 50PG; 30% PEG400; 10% ethanol – o.g.) for 21 days (3 cycles of 5 days treatment/2 days off). **D** Median fold of human leukemic cells over murine CD45^+^ cells calculated at day 41 post engraftment, pooled from two independent experiments, *n* = 6 (control), *n* = 8 (AUR), *n* = 7 (VEN, VEN+AUR), each dot represents a separate mouse. *P* values were calculated with the Mann–Whitney *U* test. **E** Event-free survival depicted on Kaplan–Meier survival plot from two independent experiments. Curve comparison was performed by log-rank (Mantel–Cox) test. **F** Experimental scheme for PDX#2. PDX#2 cells were injected into NSG mice and disease progression was determined based on peripheral blood staining. After 1 week, once leukemia cells propagation was confirmed in murine blood (0.2–1% of human leukemic cells among murine CD45^+^ cells), VEN and AUR administration was initiated. Randomly selected mice were treated with VEN, AUR, and a combination of both drugs as described in (**C**), daily, for 28 days. **G** Median fold of human leukemic cells over murine CD45^+^ cells calculated at day 25 post engraftment, *n* = 7 (control, AUR), *n* = 8 (VEN, VEN+AUR), each dot represents a separate mouse. *P* values were calculated with the Mann–Whitney *U* test. **H** Event-free survival for PDX#2 depicted on Kaplan–Meier survival plot. Curve comparison was performed by log-rank (Mantel–Cox) test; numbers of mice were as reported in (**G**).

The *TP53*-independent spectrum of AUR activity prompted us to test the cytotoxic effects of AUR in combination with VEN in MLLr BCP-ALL cells. In SEM and RS4;11 cells AUR enhanced VEN cytotoxicity, with combination indices (CI) of 0.49–0.9, indicating a synergistic interaction (Fig. 2A). Next, we tested the cytotoxic effects of VEN and AUR in MLLr BCP-ALL PDXs (Supplementary Table 1) co-cultured with primary BM-MSC [31]. AUR synergistically enhanced VEN cytotoxicity in 8/8 tested PDXs (Fig. 2B and Supplementary Fig. 4A, B). Each drug, as well as their combination, was not cytotoxic toward BM-MSC (Supplementary Fig. 4C). Moreover, we have not observed any strong enhancement of VEN cytotoxicity by AUR toward human CD19^+^-enriched cells isolated from healthy donors (Supplementary Fig. 4D). In addition, we observed an increase of VEN cytotoxicity by AUR in Ph-positive and hypodiploid BCP-ALL cell lines as well as in PDXs representing Ph-positive and Ph-like genetic subtypes (*n* = 4), and no effects toward OP9 stromal cells (Supplementary Fig. 5A–F).

Given that AUR improved VEN cytotoxicity in vitro, we aimed to test the antileukemic efficacy of the combination in vivo in MLLr BCP-ALL PDXs, derived from two different patients (at the stage of diagnosis and relapse) (Fig. 2C–H). In PDX#1, although VEN and AUR single treatments significantly attenuated leukemia progression, human BCP-ALL cells were still detectable in murine blood after the treatment and their numbers were significantly lower following the combination therapy (Fig. 2D). Consistent with the reduced BCP-ALL cell numbers, we observed a significant improvement in survival with the combination of VEN and AUR compared to any single treatment (Fig. 2E). Importantly, AUR also significantly improved VEN antileukemic efficacy in a very aggressive PDX#2 derived from MLLr pediatric patient at relapse stage (Fig. 2G, H). Altogether, these results indicate that AUR enhances VEN efficacy in vitro against various BCP-ALL subtypes and improves VEN activity against MLLr BCP-ALL in vivo.

### Functional p53 is not essential for the in vitro effectiveness of VEN and AUR combination

**Fig. 3:**
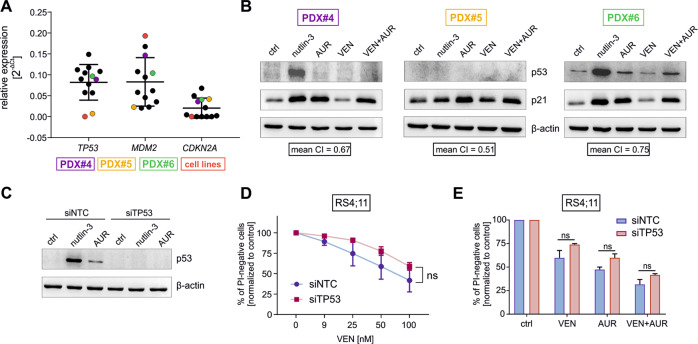
Functional p53 is not essential for VEN+AUR effectiveness in vitro. **A** mRNA expression of *TP53*, *MDM2*, and *CDKN2A* was determined by RT-qPCR in PDXs representing MLLr BCP-ALL (*n* = 8) and Ph-positive/Ph-like subtypes (*n* = 4). The graph depicts mean value ± SD, and each dot corresponds to an individual sample. The red dots represent measured data for cell lines with deletions or high expression of particular genes and serve as controls. Cell lines were selected based on data from https://www.cbioportal.org/ and [58]. Selected cell lines are as follows: HL-60 for *TP53 null*, MUTZ-5 for *MDM2* overexpression, and BV-173 for *CDKN2A null*. Colored dots represent MLLr BCP-ALL PDXs selected for (**B**), while black dots represent the remaining PDX samples. **B** Selected PDX cells were exposed to 10 μM nutlin-3, 1 μM AUR, 5–50 nM VEN, or VEN+AUR combination for 6 h and the protein levels of p53 and p21 were detected by immunoblotting. β-actin levels show the protein loading, *n* = 1. Framed numbers under each blot depict mean CI value of VEN+AUR combination for selected PDXs (see also Fig. 2B). **C** Representative immunoblot of control (siNTC) and *TP53* targeting (siTP53) siRNA-treated RS4;11 cells. 24 h post electroporation the cells were exposed to DMSO (control), 10 μM nutlin-3 or 1.5 μM AUR for an additional 6 h and p53 protein levels were evaluated, *n* = 3. **D** RS4;11 control (siNTC) cells and the corresponding cells with p53 knockdown (siTP53) were exposed to increasing concentrations of VEN for 24 h, before cells were analyzed for PI staining by flow cytometry to measure cell death. The graph represents mean values ± SD from three independent knockdown experiments. Statistical analysis was performed using a two-way ANOVA test. **E** siNTC and siTP53 RS4;11 cells were treated with DMSO, 45 nM VEN, 0.8 μM AUR, or a combination of both. Following 24 h, the percentage of dead cells was detected by PI staining and flow cytometry. The bars indicate mean + SD from two independent knockdown experiments. In each group the statistical significance was calculated by a paired *t*-test; ns not significant.

Considering that we observed attenuation of the p53 pathway in BCP-ALL cells subjected to VEN treatment, we next aimed to elucidate if the status of p53 affects the efficacy of the combination of VEN+AUR. First, we confirmed the p53 status of the PDXs analyzed. In BCP-ALL PDXs that responded synergistically to VEN+AUR (Fig. 2B and Supplementary Figs. 4 and 5) we found no point mutations of *TP53* (data not shown), which is consistent with the rare occurrence of *TP53* mutations in BCP-ALL [32]. However, other genetic defects leading to *TP53* mRNA downregulation [33] or p53 protein degradation, such as a mouse double minute 2 homolog (*MDM2*) overexpression or cyclin-dependent kinase inhibitor 2A (*CDKN2A*) deletion, are more frequently found in BCP-ALL [34]. Accordingly, we found a lack of *TP53* expression in one PDX (PDX#5), *MDM2* overexpression in two, and lack of *CDKN2A* mRNA in seven, suggesting that the activity of p53 could be impaired in these PDXs (Fig. 3A).

For further analysis of p53 functionality, we selected two MLLr BCP-ALL PDXs exhibiting p53-associated aberrations, one with *MDM2* overexpression (PDX#4) and the other showing no *TP53* expression (PDX#5). As a reference, we selected one PDX (PDX#6) showing no p53 dysfunctions (Fig. 3A). In PDX#4 and PDX#6 p53 was potently induced by nutlin-3, an inhibitor of p53-MDM2 interaction that triggers p53 accumulation, but only slightly induced by AUR and VEN+AUR, solely in PDX#6 (Fig. 3B). Following AUR and VEN+AUR treatment, we observed induction of p21, the p53 target gene and a marker of p53 activation, in all tested PDXs including PDX#5, which lacks detectable p53, indicating the ability of AUR to induce p21 in p53-independent manner (Fig. 3B). Importantly, in all three PDXs the efficacy of VEN+AUR was similar, with mean CI ranging from 0.51 to 0.75 (Fig. 2B), suggesting that AUR is able to potentiate VEN efficacy in both p53-proficient and -deficient BCP-ALL cells.

To further investigate the role of p53 in VEN+AUR efficacy, we used siRNA to downregulate p53 in RS4;11 cells, which harbor WT p53 (Fig. 3C). We observed that p53 knockdown had no significant effect on VEN response in RS4;11 cells in 24 h assay (Fig. 3D). This was also true for VEN and AUR combination (Fig. 3E). Importantly, downregulation of p53 did not perturb the ability of AUR to sensitize the cells to VEN treatment nor to induce p21 (Supplementary Fig. 6A, B). Altogether, these results indicate that the presence of functional p53 is not essential for AUR to potentiate VEN cytotoxicity in vitro toward MLLr BCP-ALL cells.

### AUR potentiates VEN-mediated apoptosis and upregulates pro-apoptotic protein NOXA

Next, we investigated the mechanism of the synergistic interaction between VEN and AUR using the SEM cell line as a model. Given that AUR inhibits cytoplasmic (TXNRD1) and mitochondrial (TXNRD2) thioredoxin reductases [35], we asked whether downregulation of these enzymes would also improve VEN efficacy. In SEM cells with siRNA-reduced levels of TXNRD1 or TXNRD2 we observed only minor sensitization to VEN (Supplementary Fig. 7A, B). TXNRD1 is a superordinate enzyme of the TXN system that scavenges cytoplasmic ROS [28], hence its inhibition by AUR increases cytoplasmic ROS levels (Supplementary Fig. 7C). However, in contrast to AUR, treatment with VEN or VEN+AUR reduced cytoplasmic ROS levels (Supplementary Fig. 7C), arguing that the combination does not trigger oxidative stress via TXNRD1 inhibition.

**Fig. 4:**
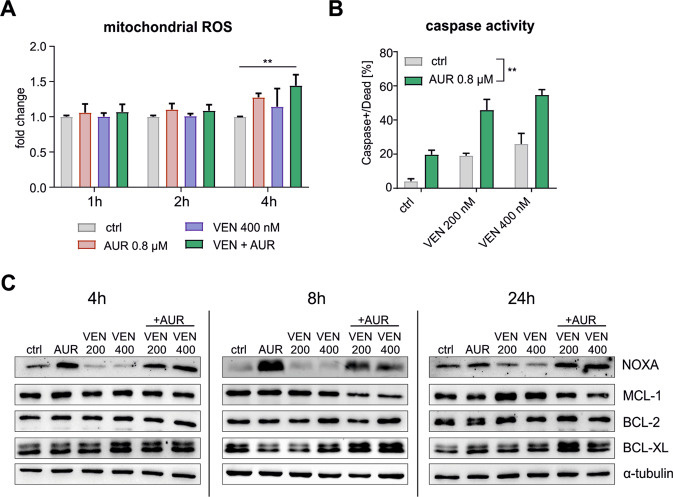
AUR potentiates apoptosis induction by VEN and strongly upregulates pro-apoptotic protein NOXA. **A** SEM cells were exposed to VEN, AUR and a combination of both drugs for 1, 2 and 4 h, and then mitochondrial ROS levels were detected by MitoSOX assay. The bars represent mean fold change in mean fluorescence intensity (MFI) over untreated control (DMSO) + SD, *n* = 4. **P < 0.01 by one-way ANOVA with Bonferroni post-hoc test. **B** SEM cells were treated with VEN, AUR, and both drugs for 16 h and subjected to MultiCaspase assay. Activation of caspases and cell death was determined by staining of the cells with fluorescently-labeled inhibitor of caspases (FLICA) and 7AAD dye and analyzed using Muse Cell Analyzer. The bars show mean values + SD (*n* = 4). Statistical analysis was performed using a two-way ANOVA test, and the significance for the interaction factor was presented, **P < 0.01. **C** SEM cells were exposed to indicated concentrations (in nM) of VEN, 0.8 μM AUR and a combination of both drugs for 4, 8, and 24 h. The levels of particular members of BCL-2 family proteins were determined by immunoblotting. The graph shows representative blots of at least two independent experiments. α-tubulin serves as a loading control.

TXNRD2 is a mitochondrial enzyme, which scavenges ROS within the matrix. We observed an increase in mitochondrial ROS levels in cells incubated with VEN+AUR (Fig. 4A), suggesting that AUR may act at the mitochondrion to potentiate VEN activity. Given that VEN triggers intrinsic mitochondrial apoptosis [36], we investigated the influence of AUR on VEN-induced apoptosis. Caspase activity was induced in response to either VEN or AUR alone, but was greatly increased in cells treated with both drugs (Fig. 4B and Supplementary Fig. 8A). In addition, we detected accumulation of cleaved PARP, indicating caspase-3 activation, a hallmark of apoptosis (Supplementary Fig. 8B). To gain further insight into the mechanism of apoptosis induction, we measured the levels of proteins of the BCL-2 family. The most striking observation was the strong upregulation of pro-apoptotic protein NOXA, seen after 4 and 8 h of incubation with AUR and VEN+AUR, but not with VEN alone, and persisting even after 24 h (Fig. 4C). In contrast, the NOXA-binding anti-apoptotic protein MCL-1 was induced upon 24 h of treatment with VEN, however, this was not observed for the combination treatment with AUR (Fig. 4C). We also observed some increase in other proteins (BCL-XL, BAK) in response to single drugs and their combination, although these changes were less pronounced (Fig. 4C and Supplementary Fig. 8C).

### Deletion of NOXA in SEM cells abrogates VEN+AUR-mediated cell death

**Fig. 5:**
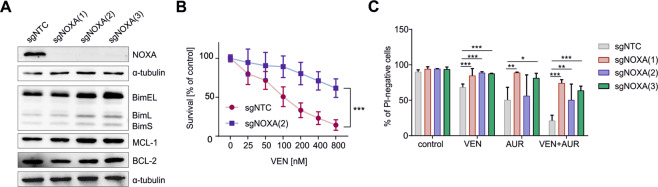
NOXA expression is functionally relevant for VEN+AUR drug combination activity. **A** The basal levels of BCL-2 family proteins were determined by immunoblotting in control SEM cells (sgNTC) and in mixed clones of CRISPR-Cas9-mediated NOXA knockout cells generated using three independent sgRNAs (sgNOXA1-3). The graph shows representative blots from two independent cell pools. **B** Control SEM cells (sgNTC) and NOXA genomic knockout cells [sgNOXA(2)] were treated with VEN for 48 h and subjected to MTT viability assay. The data are presented as mean percentage of untreated cells (DMSO) ± SD, ***P < 0.001 by two-way ANOVA for the interaction factor. **C** Control SEM cells (sgNTC) and cells with NOXA genomic knockout [sgNOXA(1-3)] were treated with VEN [60 nM for sgNOXA(1) and sgNOXA(3), 90 nM for sgNOXA(2)], AUR (0.8 μM) or both for 48 h and then the proportion of dead cells was assessed with propidium iodide (PI) staining by flow cytometry. The data are presented as mean percentage of PI-negative cells for each independent sgRNA, *n* > 3. In each group (VEN, AUR, and VEN+AUR), the difference in viability between NOXA genomic knockout and control cells was assessed by one-way ANOVA with Dunnett’s post-hoc test, *P < 0.05, **P < 0.01, ***P < 0.001.

To evaluate the functional role of NOXA in cytotoxic effects of VEN+AUR, we generated SEM cells with a CRISPR-Cas9-mediated knockout of the NOXA-encoding gene, *PMAIP1*. Three different *PMAIP1–*targeting sgRNAs resulted in undetectable NOXA protein (Fig. 5A). This correlated with increased MCL-1, consistent with a role for NOXA in MCL-1 degradation (Fig. 5A) [37]. Importantly, the absence of NOXA significantly diminished the cytotoxicity of VEN, AUR, and the VEN+AUR combination (Fig. 5B, C), despite marked upregulation of pro-apoptotic BIM (Fig. 5A). Conversely, the absence of NOXA only slightly reduced the cytotoxicity of doxorubicin or vincristine, indicating a drug-specific role of NOXA in mediating cell death (Supplementary Fig. 9).

### AUR promotes potent transcriptional induction of *PMAIP1* gene

**Fig. 6:**
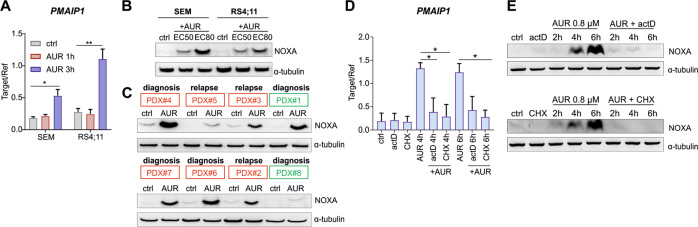
AUR triggers transcriptional induction of NOXA-encoding gene in MLLr BCP-ALL cell lines and PDXs. **A** mRNA expression of NOXA-encoding gene, *PMAIP1*, in MLLr BCP-ALL cell lines exposed to EC50 concentration of AUR for 1 and 3 h. Data are presented as the mRNA level of *PMAIP1* relative to *RPL29* and *GUSB* control genes. Bars show mean values + SD, *n* = 2. *P < 0.05, **P < 0.01 by one-way ANOVA with Dunnett’s post-hoc test. **B** MLLr BCP-ALL cell lines were treated with EC50 or EC80 of AUR and collected after 8 h. NOXA protein levels were assessed by immunoblotting. The graph is representative of two independent experiments. **C** MLLr BCP-ALL PDXs were exposed to 1.5 μM AUR for 4 h and NOXA level was determined by immunoblotting, *n* = 1. Red frames indicate PDX samples generated from pediatric, while green from adult patients. **D**, **E** SEM cells were pre-incubated with 1 µg/ml actinomycin D (actD) or 50 µg/ml cycloheximide (CHX) for 15 min and then treated with EC50 concentration of AUR (0.8 μM) for 2, 4, and 6 h. The mRNA levels of *PMAIP1* gene and NOXA protein levels were evaluated by RT-qPCR and immunoblotting, respectively. The graph depicts the mean mRNA level of *PMAIP1* gene relative to control genes (*RPL29*, *GUSB*) + SD from two independent experiments, *P < 0.05 by one-way ANOVA with Bonferroni post-hoc test. Only selected significantly different groups are indicated in the graph (**D**). Representative immunoblots illustrate NOXA protein level, *n* = 2 (**E**); α-tubulin serves as a loading control in **B**, **C** and **E**.

Given the key role of NOXA in the efficacy of VEN+AUR combination, we tested the effects of AUR on NOXA expression in a range of BCP-ALL cell lines. We observed an increase in NOXA-encoding mRNA in response to AUR (Fig. 6A) and concentration-dependent accumulation of NOXA protein in MLLr BCP-ALL cell lines (Fig. 6B), PDXs (*n* = 8, Fig. 6C), as well as in other BCP-ALL cell lines (Supplementary Fig. 10A, B). In contrast, AUR did not cause strong upregulation of NOXA protein in human PBMC isolated from healthy donors (Supplementary Fig. 10C). To further investigate the mechanism of NOXA induction, we checked the effects of inhibiting global transcription (actinomycin D, actD) and translation (cycloheximide, CHX) on the ability of AUR to induce NOXA in SEM cells. Both actD and CHX almost completely abrogated the increase of *PMAIP1* mRNA (Fig. 6D) and blocked the accumulation of NOXA protein in response to AUR (Fig. 6E). These results indicate that AUR induces *PMAIP1* gene transcription and subsequent accumulation of NOXA protein in BCP-ALL cells.

### AUR drives increased chromatin accessibility at *PMAIP1*

**Fig. 7:**
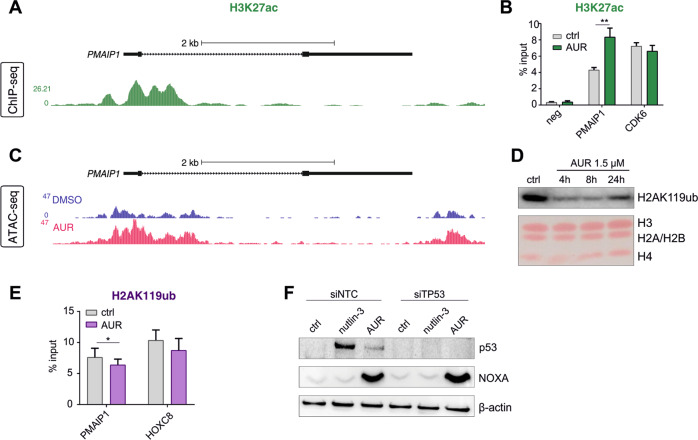
AUR-mediated induction of NOXA-encoding *PMAIP1* gene transcription is associated with chromatin remodeling. **A** H3K27ac ChIP-seq track derived from untreated SEM cells (taken from GSE74812). **B** SEM cells were treated with either DMSO (control) or with 1.5 μM AUR (EC80) for 4 h and H3K27ac enrichments at *PMAIP1, CDK6* (positive control) and negative control (neg) regions were determined by ChIP-qPCR. The graph depicts mean values + SD from four independent biological repeats (*n* = 4). **P < 0.01 by a paired *t*-test. **C** ATAC-seq at *PMAIP1* in SEM cells treated with either DMSO (control, blue peaks) or 1.5 μM AUR (pink peaks) for 4 h. **D** SEM cells were exposed to AUR and a nuclear fraction of the cells was collected after an indicated period of time. Histone H2A monoubiquitination at K119 (H2AK119ub) was detected by immunoblotting, whereas Ponceau staining was performed to assess equal protein loading. Immunoblot is a representative of two independent experiments. **E** SEM cells were treated with either DMSO (control) or with 1.5 μM AUR (EC80) for 4 h and H2AK119ub enrichments at *PMAIP1* and *HOXC8* (positive control) regions were determined by ChIP-qPCR. The graph depicts mean values + SD from four independent biological repeats (*n* = 4). *P < 0.05 by a paired *t*-test. **F** 24 h following siRNA-mediated p53 downregulation, SEM cells were exposed to DMSO (control), 10 μM nutlin-3 or 1.5 μM AUR for 6 h and p53 and NOXA protein levels were evaluated by immunoblotting; immunoblot is a representative of three independent experiments.

To understand the mechanism by which AUR induces *PMAIP1* expression, we analyzed the chromatin environment of the gene. We found that the promoter of *PMAIP1* is marked by histone H3 lysine-27 acetylation (H3K27ac) even in untreated SEM cells (Fig. 7A), suggesting that the gene is poised for activation. Upon AUR treatment, there was a significant increase in H3K27ac levels at *PMAIP1* (Fig. 7B), correlating with activation of the gene. Next, using ATAC-sequencing (ATAC-seq), we asked whether this was associated with a change in chromatin accessibility. Strikingly, we observed a strong increase in accessibility following AUR treatment, suggesting that gene activation is associated with chromatin remodeling at this locus (Fig. 7C). Treatment with AUR also decreased the repressive modification—histone H2A lysine-119 monoubiquitylation (H2AK119ub)—in nuclear lysates (Fig. 7D) as well as to some extent also at the *PMAIP1* locus (Fig. 7E). As H3K27ac increases and enhanced ATAC signal are associated with gene activation, and decreases in H2AK119ub are associated with reduced gene repression, these chromatin changes are consistent with activation of *PMAIP1*.

The lack of increase in *PMAIP1* mRNA in response to AUR in SEM cells pre-incubated with CHX may suggest a requirement for the translation of upstream transcriptional regulators in *PMAIP1* expression that are induced by AUR treatment. Although NOXA is one of the key transcriptional targets of p53 [38], in SEM cells a role for p53 in NOXA upregulation in response to AUR is unlikely as *TP53* is mutated. Moreover, downregulation of p53 in RS4;11 cells, harboring wild-type *TP53*, had no impact on the upregulation of NOXA expression in response to AUR (Fig. 7F), indicating that p53 is not involved in AUR-mediated induction of NOXA. Recently, 5-azacytidine has been shown to induce *PMAIP1* gene transcription via the integrated stress response (ISR) pathway [39]. We have previously demonstrated that AUR triggers canonical ISR elements in BCP-ALL cells, such as BIP and CHOP induction as well as phosphorylation of eIF2α [28]. Therefore, we aimed to investigate the role of ATF4, the main effector of ISR, in NOXA upregulation by AUR. In SEM cells incubated with AUR or the classical endoplasmic reticulum stress inducer tunicamycin (TUNI), we observed strong induction of ATF4 (Supplementary Fig. 11A) and found ATF4 bound to *PMAIP1* (Supplementary Fig. 11B). However, despite the very effective siRNA-mediated downregulation of ATF4, it only slightly reduced NOXA induction by AUR, indicating the involvement of other factors (Supplementary Fig. 11C, D). In line with these data, TUNI treatment only slightly induced NOXA expression, further confirming that ISR itself is insufficient for the potent NOXA upregulation observed in response to AUR in SEM cells (Supplementary Fig. 11A, E). This also suggests that despite the increased binding, ATF4 is not the major regulator of *PMAIP1* expression under AUR induction conditions. Taken together, these results show that AUR treatment leads to chromatin remodeling at the *PMAIP1* locus, associated with the transcriptional activation of this gene. AUR-mediated NOXA induction is p53-independent and is regulated by as-yet-unidentified transcription factors, but is partially regulated by ATF4 and ISR.

## Discussion

The significance of employing VEN in the treatment of MLLr BCP-ALL, a leukemia subtype for which there is no curative treatment, has been highlighted in several studies [18, 21, 40]. However, insufficient preclinical responses to VEN as monotherapy [18, 21, 41] prompt a need for optimal drugs for combination therapy. To better select a drug that could improve VEN antileukemic activity in MLLr BCP-ALL we investigated alterations in gene expression in leukemic cells subjected to VEN in vivo by RNA-seq. We found significant differences in the expression of the p53 pathway-associated genes. The significance of intact p53 for VEN efficacy has been extensively studied in recent years, revealing lineage-dependent results. In CLL, VEN exerted similar cytotoxic effects in p53 wild-type and mutant cells and worked effectively in patients with *TP53* mutations [14, 15]. Conversely, studies conducted in AML revealed that blasts with *TP53* mutations are less sensitive to VEN and that inactivation of *TP53* or its pro-apoptotic targets (*BAX*, *PMAIP1*) confers resistance to VEN [42]. In hypodiploid BCP-ALL, intact p53 was important for the cytostatic but not the cytotoxic effect of VEN [20]. In this study, in MLLr BCP-ALL PDXs subjected to VEN for about 2 weeks of in vivo treatment, we found downregulation of p53 target genes involved in apoptosis, cell cycle arrest as well as upregulation of negative-p53 regulators, indicating impairment of the p53 pathway in response to VEN. Presumably, these changes may result from the selection of clones differentially expressing these genes under VEN treatment, however, further studies are needed to elucidate the exact mechanism. These findings are in line with a recent report showing that mantle cell lymphoma (MCL) patients acquire *TP53* mutations upon progression on VEN [43]. Deletions of *CDKN2A*, which encodes a p53 activator p14ARF, were found in CLL [44] and MCL patients [43] who relapsed after VEN, implying association of the p53 pathway with VEN resistance. Also, as recently shown, p53-deficient leukemias outcompeted corresponding controls with intact *TP53* to escape VEN treatment [45].

Considering the impairment of p53-dependent apoptosis by VEN in MLLr BCP-ALL, we selected AUR, a drug that had been reported to exert cytotoxic effects independently of p53 [24], for the combination treatment with VEN. AUR killed MLLr BCP-ALL cell lines with wild-type (RS4;11) and mutant (SEM) p53 with similar efficacy and enhanced VEN activity in both cell lines. AUR also enhanced antileukemic activity of VEN in two MLLr BCP-ALL PDXs in vivo, leading to improved survival of combination-treated leukemic mice. Importantly, the in vitro co-treatment with VEN and AUR was also synergistic in other BCP-ALL subtypes such as hypodiploid, Ph-positive, and Ph-like, indicating that the synergistic effect is not specific for the MLLr BCP-ALL. While this suggests that AUR may also be a candidate for combination with VEN in other HR BCP-ALL subtypes, more research is needed to assess the efficacy of this combination in vivo and better understand the mechanism in non-MLLr subtypes.

Notably, the in vitro activity of the combination of AUR and VEN did not require functional p53. Unlike in solid tumors, direct alterations in *TP53* gene, either somatic mutations or deletions, are rare in BCP-ALL at diagnosis but are acquired during treatment or at relapse [32, 46]. In our cohort of 12 BCP-ALL patients, we detected no *TP53* point mutations. However, we found other genetic aberrations that functionally impair the p53 pathway such as lack of *TP53* mRNA, *MDM2* overexpression, and *CDKN2A* deletions. Importantly, in all cases interaction between VEN and AUR was synergistic. The efficacy of VEN and AUR did not differ between RS4;11 cells with or without functional p53, indicating a lack of p53 dependence. To conclude, these results indicate that drugs, such as AUR, working independently of p53 are worth further preclinical testing in combination with VEN.

The role of specific pro-apoptotic BCL-2 family members in the induction of apoptosis is well understood [10]. Here, we show that AUR mediates a robust increase of pro-apoptotic protein NOXA. Importantly, SEM cells lacking NOXA were significantly less sensitive to VEN, AUR, and VEN+AUR combination, highlighting the functional relevance of NOXA expression in apoptosis in vitro. NOXA preferentially binds anti-apoptotic protein MCL-1 and promotes its degradation [47, 48]. MCL-1 was shown as one of the key mediators of resistance to VEN in various hematological cancers [49–52]. We can speculate that in MLLr BCP-ALL, NOXA induction by AUR may prevent the anti-apoptotic function of MCL-1. Indeed, in SEM cells incubated with VEN we observed the induction of the MCL-1 protein, which was prevented by the addition of AUR. Pharmacologic inhibition of MCL-1 has already been presented as a good strategy to improve VEN efficacy in various hematological cancers [22, 49, 50], however, important safety concerns have been recently raised [53]. Considering that MCL-1 inhibitors are not yet approved for clinical use and further studies are needed to confirm their safety in humans, MCL-1 inactivation by AUR-mediated induction of NOXA may serve as an alternative approach. Consistent with our observation, induction of NOXA has been recently described as the main driver of synergistic activity of 5-azacytidine and VEN, a drug combination that is approved for the treatment of AML [39, 54]. BIM, another pro-apoptotic protein, was found to be crucial for potentiation of VEN efficacy by dexamethasone and imatinib in Ph-positive BCP-ALL models [19]. In conclusion, the above data suggest that the induction of various pro-apoptotic proteins is of key importance for increasing the efficacy of VEN.

AUR is widely known as an inhibitor of TXNRD1/2 antioxidant enzymes [35], yet other targets have also been reported [55]. We did not observe any strong effect of TXNRD1/2 downregulation on VEN activity. To further elucidate how AUR potentiates VEN cytotoxicity we focused on the mechanism of NOXA accumulation by AUR. For the first time, we present AUR as a drug that promotes epigenetic changes and chromatin relaxation in the NOXA-encoding gene, making it more susceptible to transcriptional activation. We note that it is unclear whether this chromatin remodeling is a cause or consequence of transcription factor binding at the gene following AUR treatment. Interestingly, the upregulation of NOXA was p53-independent and only partially dependent on ATF4, the key mediator of ISR. Several other transcription factors have been reported to be engaged in the regulation of *PMAIP1* gene expression including c-MYC [56], thus additional studies are needed to elucidate the role of other transcriptional regulators of NOXA induction by AUR.

In conclusion, this study has identified AUR as a promising candidate to be combined with VEN to improve its antileukemic activity in MLLr BCP-ALL. AUR is a registered drug used in rheumatoid arthritis with antitumor properties that can be repurposed for other indications. Given that VEN+AUR combination works independently of p53 status and that *TP53* mutations are important prognostic parameters for BCP-ALL patients, it may be beneficial to include AUR in novel, VEN-containing drug combinations tested for the efficacy in R/R BCP-ALL patients with *TP53* mutations.

## Materials and methods

### Animal studies testing VEN+AUR antileukemic activity

Approval for the experiments was given by the Ethics Committee of the Medical University of Warsaw (639/2018).

Cryopreserved MLLr BCP-ALL PDXs were thawed and injected intravenously to the tail vein of male and female 4–8 weeks old NOD.Cg-Prkdc^scid^ Il2rg^tm1Wjl^/SzJ (NSG) mice (Charles River Laboratories, Wilmington, MA, USA), 0.5 × 10^6^ cells per mouse. Cells engraftment was monitored weekly in the murine peripheral blood (collected from the cheek vein) by determination of human CD45^+^, CD19^+^ cells among murine CD45^+^ leukocytes using specific antibodies (Supplementary Table 2) and flow cytometry. The percentage of human cells was presented as (hCD45^+^hCD19^+^/mCD45^+^) × 100%. Confirmed engraftment of the leukemic cells at the minimum level of 1% (PDX#1) or 0.2% (PDX#2) in all mice was the starting point of the drug administration (day 18 post cells injection for PDX#1 and day 7 for PDX#2). Randomly selected mice were treated with 100 mg/kg of VEN (by oral gavage, o.g), 10 mg/kg of AUR (intraperitoneally, i.p), combination of both drugs, or control (DMSO – i.p, mixture of 60% Phosal 50PG; 30% PEG400; 10% ethanol – o.g) for 3 weeks (3 cycles of 5 days on/2 days off, altogether 15 doses) for PDX#1 and for 28 consecutive days for PDX#2. Once or twice a week the leukemia burden was determined in peripheral blood as described above. The blinding was applied during outcome assessment (analysis of % of leukemic cells in peripheral blood). The mice were sacrificed when a five-fold increase of human leukemic cells to murine leukocytes was observed in two consecutive measures, or when mice displayed visible signs of illness. This includes spleen enlargement, weight loss or hind paw reflex loss.

The description of all remaining methods is provided in the Supplemental Material.

## Supplementary information

Supplementary material
