# Supplementary material for: Potent, p53-independent induction of NOXA sensitizes MLL-rearranged B-cell acute lymphoblastic leukemia cells to venetoclax
Source: Oncogene. 2022 Jan 28;41(11):1600–9. doi: 10.1038/s41388-022-02196-y (PMC8913358; doi:10.1038/s41388-022-02196-y)
Supplement: Supplementary file 1 — Supplementary material [file 41388_2022_2196_MOESM1_ESM.pdf]

Potent, p53-independent Induction of NOXA Sensitizes MLL-rearranged B Cell Acute Lymphoblastic Leukemia Cells to Venetoclax

Klaudyna Fidyt[1,2], Agata Pastorczak[3], Julia Cyran[1], Nicholas T. Crump[4], Agnieszka Goral[1], Joanna Madzio[3], Angelika Muchowicz[1], Martyna Poprzeczko[1,5], Krzysztof Domka[1,5], Lukasz Komorowski[1,2], Magdalena Winiarska[1], Joe R. Harman[4], Karolina Siudakowska[1], Agnieszka Graczyk-Jarzynka[1,5], Elzbieta Patkowska[6], Ewa Lech-Maranda[6], Wojciech Mlynarski[3], Jakub Golab[1], Thomas A. Milne[4], and Malgorzata Firczuk[1, #]

[1]Department of Immunology, Medical University of Warsaw, Warsaw, Poland

[2]Postgraduate School of Molecular Medicine, Medical University of Warsaw, Warsaw, Poland

[3]Department of Pediatrics, Oncology and Hematology, Medical University of Lodz, Lodz, Poland

[4]Medical Research Council Molecular Haematology Unit at the University of Oxford, Oxford, United Kingdom

[5]Mossakowski Medical Research Institute, Polish Academy of Sciences, Warsaw, Poland

[6]Department of Hematology, Institute of Hematology and Transfusion Medicine, Warsaw, Poland

[#]corresponding author: Malgorzata Firczuk, email: mfirczuk@wum.edu.pl, phone: +48 22 599 21 98, Department of Immunology, Medical University of Warsaw, Nielubowicza 5 Street, 02-097 Warsaw, Poland

Description: This file contains Supplementary Figures, Supplementary Materials and Methods, and Supplementary Tables.

Supplementary Figures

Sup. Fig. 1

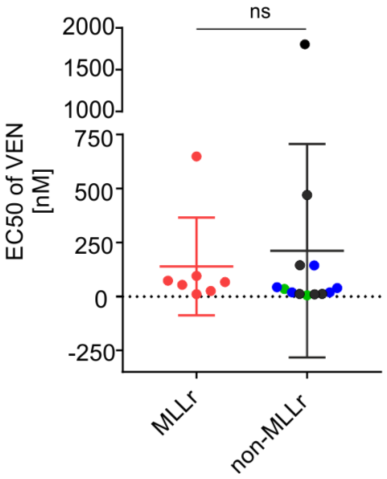

Supplementary Fig. 1. Sensitivity of primary and primograft (PDX) BCP-ALL cells to VEN. Primary cells derived from MLLr (n=7) and non-MLLr (n=13) BCP-ALL patients' bone marrow and PDX samples were incubated with VEN in monoculture for 3 days. Following drug exposure, numbers of viable cells ($CD19^+$ $7AAD^-$) were detected by flow cytometry using CountBright™ Absolute Counting Beads. EC50 value of VEN for each PDX sample was calculated by nonlinear regression dose-response analysis using GraphPad7. For non-MLLr samples, colors indicate: black – adult and pediatric BCP-ALL assigned to a standard-risk group, blue – Ph-positive or Ph-like, green – adult BCP-ALL assigned to a high-risk group with no recurrent genetic aberrations detected. Ns – not significant difference by unpaired t-test with Welch's correction.

Sup. Fig. 2

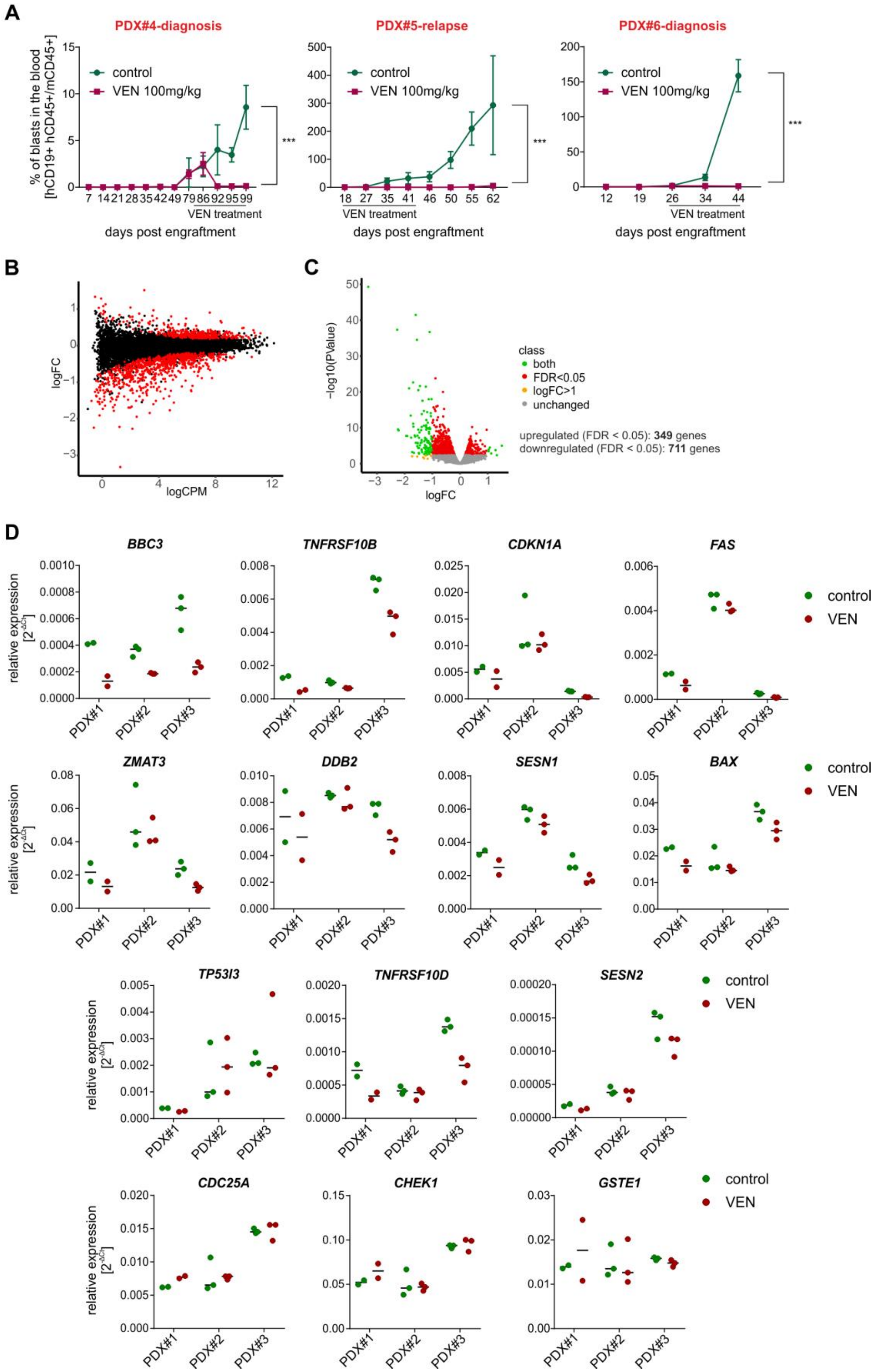

A
PDX#4-diagnosis
PDX#5-relapse
PDX#6-diagnosis
% of blasts in the blood [hCD19+ hCD45+/mCD45+]
control
VEN 100mg/kg
***
VEN treatment
days post engraftment
B
logFC
logCPM
C
-log10(PValue)
logFC
class
both
FDR<0.05
logFC>1
unchanged
upregulated (FDR < 0.05): 349 genes
downregulated (FDR < 0.05): 711 genes
D
BBC3
TNFRSF10B
CDKN1A
FAS
ZMAT3
DDB2
SESN1
BAX
TP53I3
TNFRSF10D
SESN2
CDC25A
CHEK1
GSTE1
relative expression [2^-ΔCt]
PDX#1
PDX#2
PDX#3
control
VEN

Supplementary Fig. 2. Long-term *in vivo* exposure of MLLr PDX cells to VEN induces global changes in gene expression and dysregulation of the p53 pathway genes. A, MLLr PDX cells PDX#4, PDX#5, and PDX#6 were injected into NSG mice and leukemia progression was monitored as described in Fig. 1B. Once the engraftment was confirmed in murine peripheral blood, 100 mg/kg VEN was administered 5 days per week for a total of 12 doses (PDX#4), 20 doses (PDX#5), and 15 doses (PDX#6). Graphs present the mean percentage of human blasts relative to murine $mCD45^+$ cells ± SD (n=3 mice per group for PDX#4 and PDX#6, n=7 mice per group for PDX#5). Statistical significance of the interaction effect was estimated by a 2-way ANOVA test, ***$P < 0.001$. **B,** MA plot visualizing the interaction between logFC and logCPM in RNA-seq from PDX#1 cells treated with vehicle or VEN *in vivo* with a total of 13 VEN doses (in cycles 5 days treatment/2 days off). Red dots represent differentially expressed genes (DEGs). **C,** Volcano plot showing DEGs in PDX#1 cells treated with VEN or vehicle as described in **B.** Green dots represent genes with |logFC| > 1 and FDR < 0.05; orange dots show genes with |logFC| > 1 and FDR > 0.05, whereas red dots show genes with |logFC| < 1 and FDR < 0.05. **D,** PDX#1, PDX#2, and PDX#3 were treated *in vivo* with VEN as described in Fig. 1B. RNA was isolated from $hCD19^+$-enriched cells. The graphs show the relative mRNA levels ($2^{-\Delta Ct}$) for the p53 pathway genes, as defined in Fig. 1D, assessed by RT-qPCR using gene-specific TaqMan probes, in VEN-treated and control groups. Each dot represents a separate mouse and the horizontal lines indicate the median values.

Sup. Fig. 3

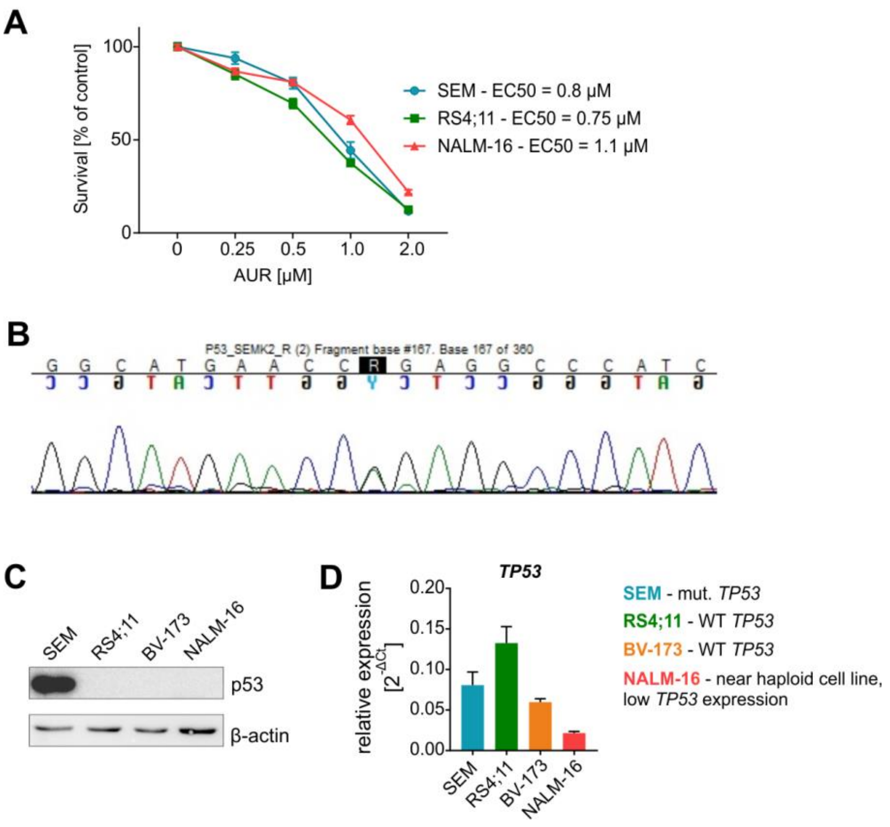

Supplementary Fig. 3. AUR kills BCP-ALL cells independently of their p53 status. A, MLLr BCP-ALL (SEM, RS4;11) and hypodiploid (NALM-16) cell lines were exposed to increasing concentration of AUR for 48 h and subjected to MTT viability assay. The graph shows mean value ± SEM from 2 independent experiments for all cell lines. Nonlinear regression dose–response analysis (GraphPad7) was used to calculate EC50 values. **B,** Sanger sequencing chromatogram depicts heterozygous *TP53* point mutation (R288Q) in SEM cell line. **C,** The basal p53 protein level was detected by immunoblotting in a panel of BCP-ALL cell lines. Equal protein loading was assessed by determination of β-actin levels. **D,** Basal *TP53* mRNA expression in BCP-ALL cell lines as determined by RT-qPCR. For each cell line mean relative expression ($2^{-\Delta Ct}$) + SD is presented from 3 biological repeats, n=3. *B2M* and *ACTB* were used as reference genes.

Sup. Fig. 4

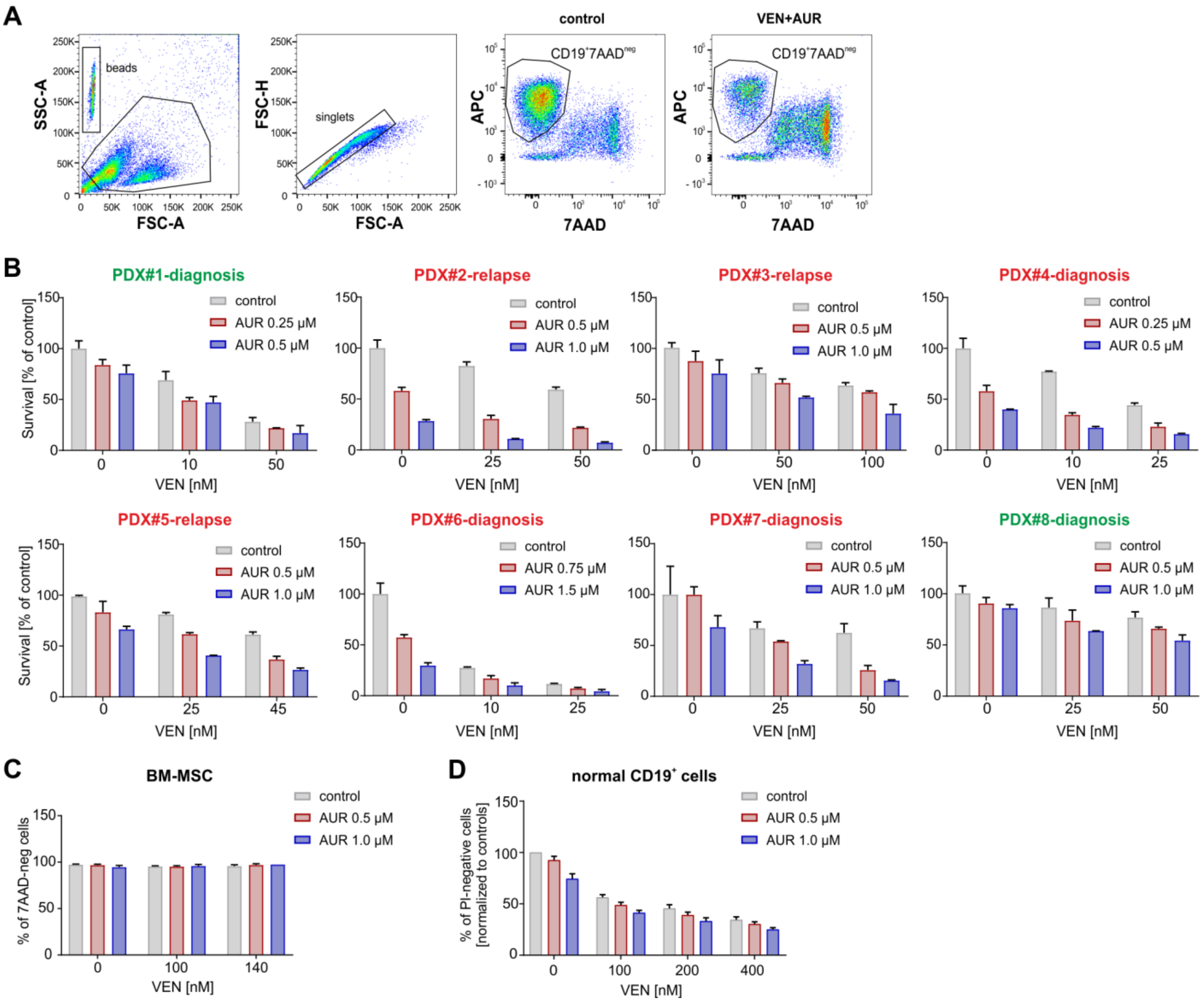

Supplementary Fig. 4. Synergistic interaction between VEN and AUR against MLLr BCP-ALL PDX cells co-cultured with BM-MSC. A, Gating strategy used for determination of viable ($CD19^{+}$ $7AAD^{-}$) cells in response to VEN, AUR, or both drugs in co-culture with BM-MSC. CountBright™ Absolute Counting Beads were used to assess the total number of leukemic cells. **B,** MLLr BCP-ALL PDXs derived from 5 diagnostic and 3 relapsed patient samples (samples derived from children marked in red and from adults marked in green) were co-cultured with primary BM-MSC cells and exposed to indicated concentrations of VEN, AUR or combination of both for 4-5 days. After that time the total number of live cells was assessed by flow cytometry. The graphs show the mean percentage of viable cells relative to control + SD from 2 wells of independently treated cells. **C,** Primary BM-MSC cells were exposed to a particular concentration of VEN, AUR, or mixture of both for 5 days. The assessment of dead cells following drug exposure was performed by flow cytometry and 7AAD staining. The bars indicate the mean percentage of 7AAD-negative cells + SEM, n=4. **D,** Human $CD19^{+}$ cells were isolated from buffy coats of 3 different healthy donors and exposed to VEN, AUR, or mixture of both for 2 days. The viability was assessed by flow cytometry as the % of propidium iodide (PI)-negative cells. The bars indicate the mean percentage of viable cells (PI-negative cells) relative to controls + SEM, n=3.

Sup. Fig. 5

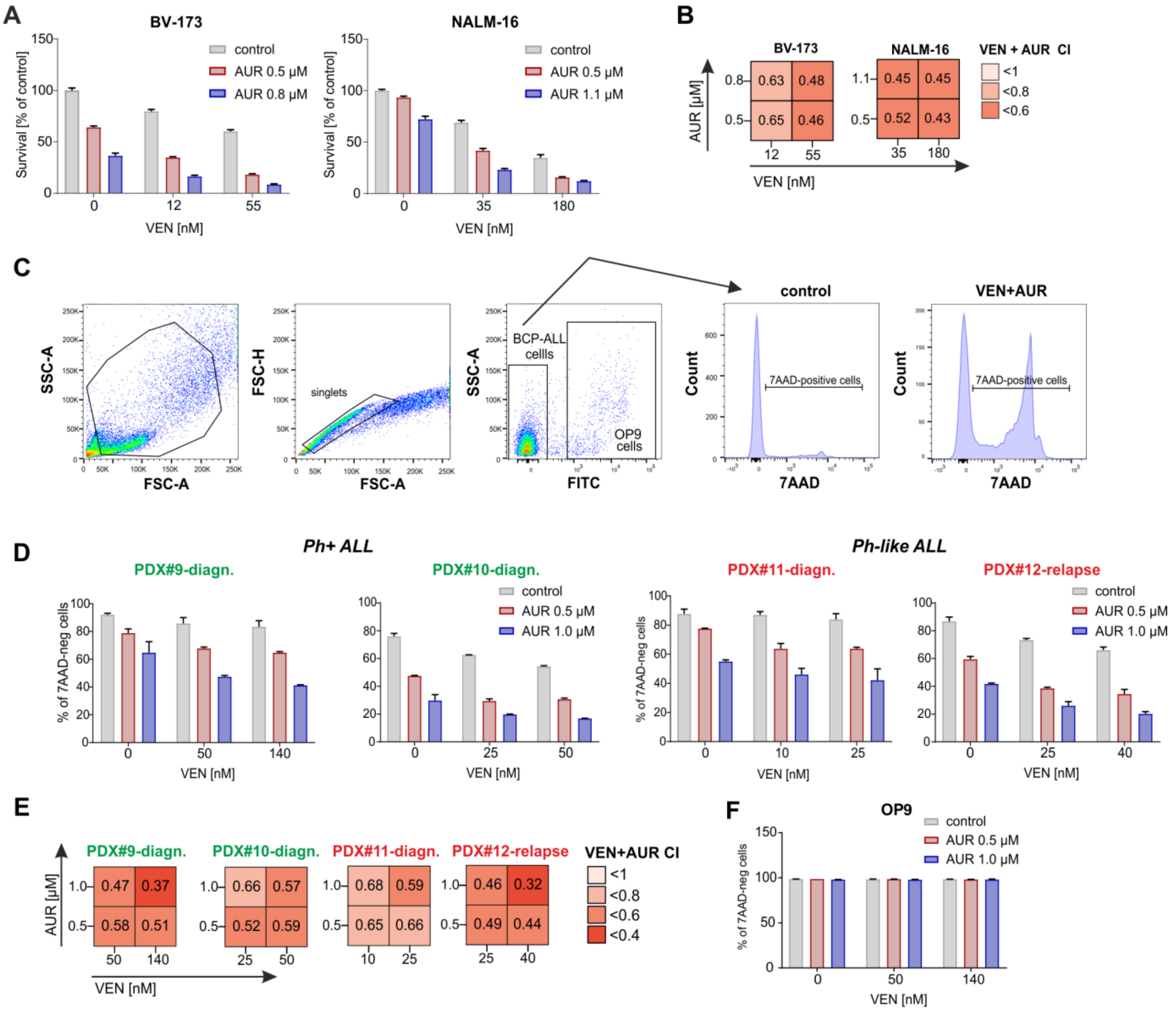

Supplementary Fig. 5. AUR potentiates VEN anti-leukemic activity against non-MLLr BCP-ALL cell lines and PDXs. A, BCP-ALL cell lines BV-173 (Philadelphia-positive, Ph+) and NALM-16 (hypodiploid) were treated with EC20 and EC50 concentrations of AUR or VEN (calculated by nonlinear regression dose-response analysis using GraphPad7), as well as with a combination of both drugs. After 48 h incubation, cells were subjected to MTT viability assay. Survival in all groups was calculated as the percentage of the controls treated with vehicle only (DMSO). Bars represent means + SEM. **B,** CI matrices representing particular VEN+AUR CI values are shown for each cell line. The combination index (CI) was calculated using CompuSyn software employing the Chou-Talalay algorithm. CI below 1 indicates synergistic interactions. The intensity of the color determines the potency of the synergistic effects. **C,** Gating strategy applied for the evaluation of the viability of BCP-ALL PDX cells co-cultured with GFP-positive OP9 stromal cells. **D,** Adult (marked in green color) and pediatric (marked in red color) Philadelphia-positive (Ph+) and Philadelphia-like (Ph-like) PDXs were co-cultured with OP9-GFP stromal cell line and treated with single drugs or VEN+AUR combination for 3 days. Cell viability was determined by 7AAD staining and flow cytometry. The bars

represent the mean percentage of 7AAD-negative cells, from 2 wells of independently treated cells + SD. **E,** Matrices illustrating VEN+AUR CI for indicated PDXs. CI below 1 indicates synergistic interactions. Colour intensity corresponds to the potency of the synergistic effects, according to legends. **F,** GFP-positive OP9 cells were exposed to VEN, AUR, or a mixture of both for 3 days. The assessment of dead cells following drugs exposure was performed by flow cytometry and 7AAD staining. The bars indicate the mean percentage of 7AAD-negative cells + SEM, n=2-4.

Sup. Fig. 6

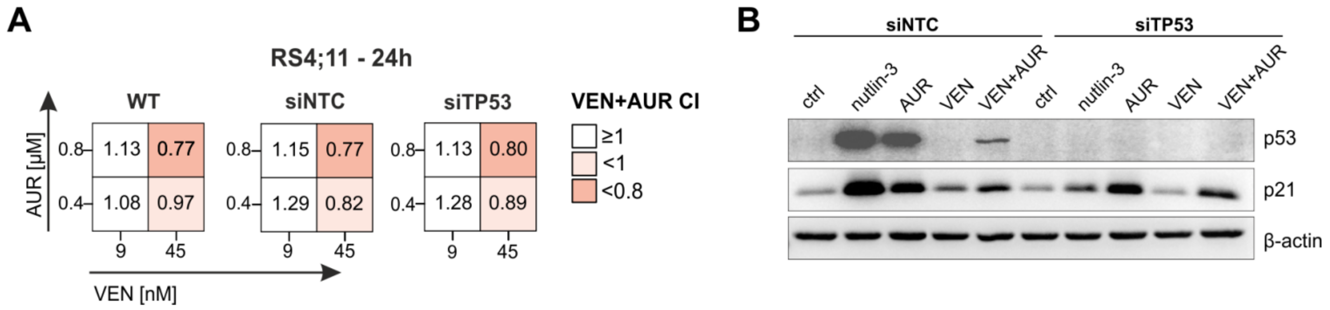

Supplementary Fig. 6. Effects of p53 downregulation on VEN+AUR efficacy and p21 induction upon AUR and VEN+AUR co-treatment. A, RS4;11 cells, either wild type (WT, n=1), siNTC (n=2) or with downregulation of p53 (siTP53, n=2) were treated with indicated concentrations of VEN, AUR, and a combination of both for 24 h. The number of dead cells was evaluated by assessment of PI-positive cells in flow cytometry. The matrices present CI which was calculated in CompuSyn software. CI < 1 indicates synergistic interaction between tested drugs. Colour intensity determines the potency of the synergistic effects, according to legends. **B,** Immunoblotting showing p53 and p21 protein levels in RS4;11 cells, either with siRNA mediated knockdown of *TP53* (siTP53) or control (siNTC), after 6 h exposure to 10 μM nutlin-3, 1.5 μM AUR, 50 nM VEN and combination of VEN and AUR. All blots are representative of 2 independent knockdown experiments.

Sup. Fig. 7

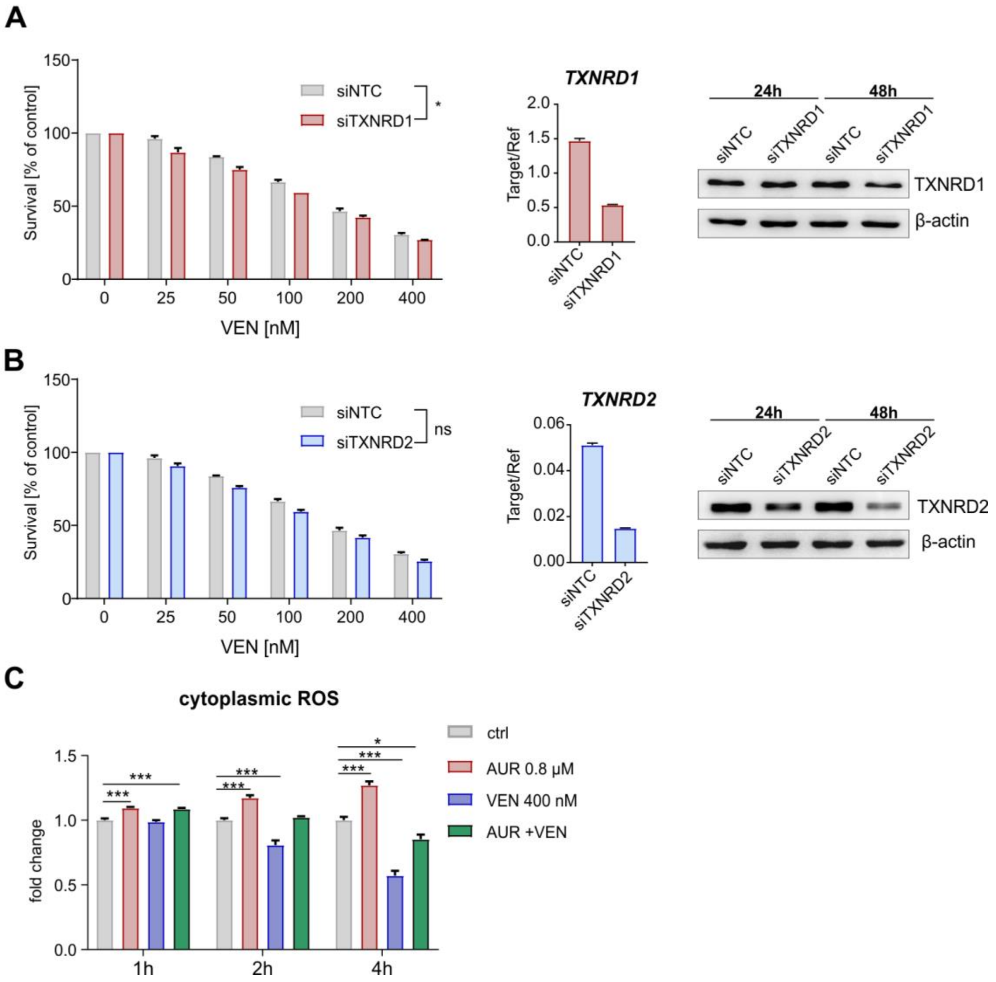

Supplementary Fig. 7. Effects of TXNRD1 and TXNRD2 downregulation on VEN cytotoxicity and cytoplasmic ROS levels in cells incubated with VEN, AUR, and VEN+AUR. A, B, Control SEM cells (siNTC) and cells with siRNA-mediated knockdown of TXNRD1 (siTXNRD1) or TXNRD2 (siTXNRD2) were treated with increasing concentrations of VEN for 48 h, and then the viability was determined by MTT assay. The data are presented as means from 2 independent knockdown experiments + SEM. *P < 0.05, ns – not significant by 2-way ANOVA test (left panels) for the interaction presented, n=2. The efficiency of siRNA-mediated knockdown was determined by RT-qPCR and immunoblotting. The graphs in the middle panel show mean mRNA expression of either *TXNRD1* or *TXNRD2* relative to control genes (*GUSB*, *RPL29*) + error. Representative immunoblots show TXNRD1 and TXNRD2 protein levels from 2 independent knockdown experiments. β-actin serves as a loading control. **C,** SEM cells were treated with indicated doses of VEN, AUR, or both drugs for 1, 2, and 4 h. After the treatment cytoplasmic ROS levels were detected by CM-H2-DCFDA dye using flow cytometry. The graph shows mean fold change in mean fluorescence intensity (MFI) over untreated control (DMSO) + SEM, n=4. *P < 0.05, **P < 0.01, ***P < 0.001 by 1-way ANOVA with Dunnett's post-hoc test.

Sup. Fig. 8

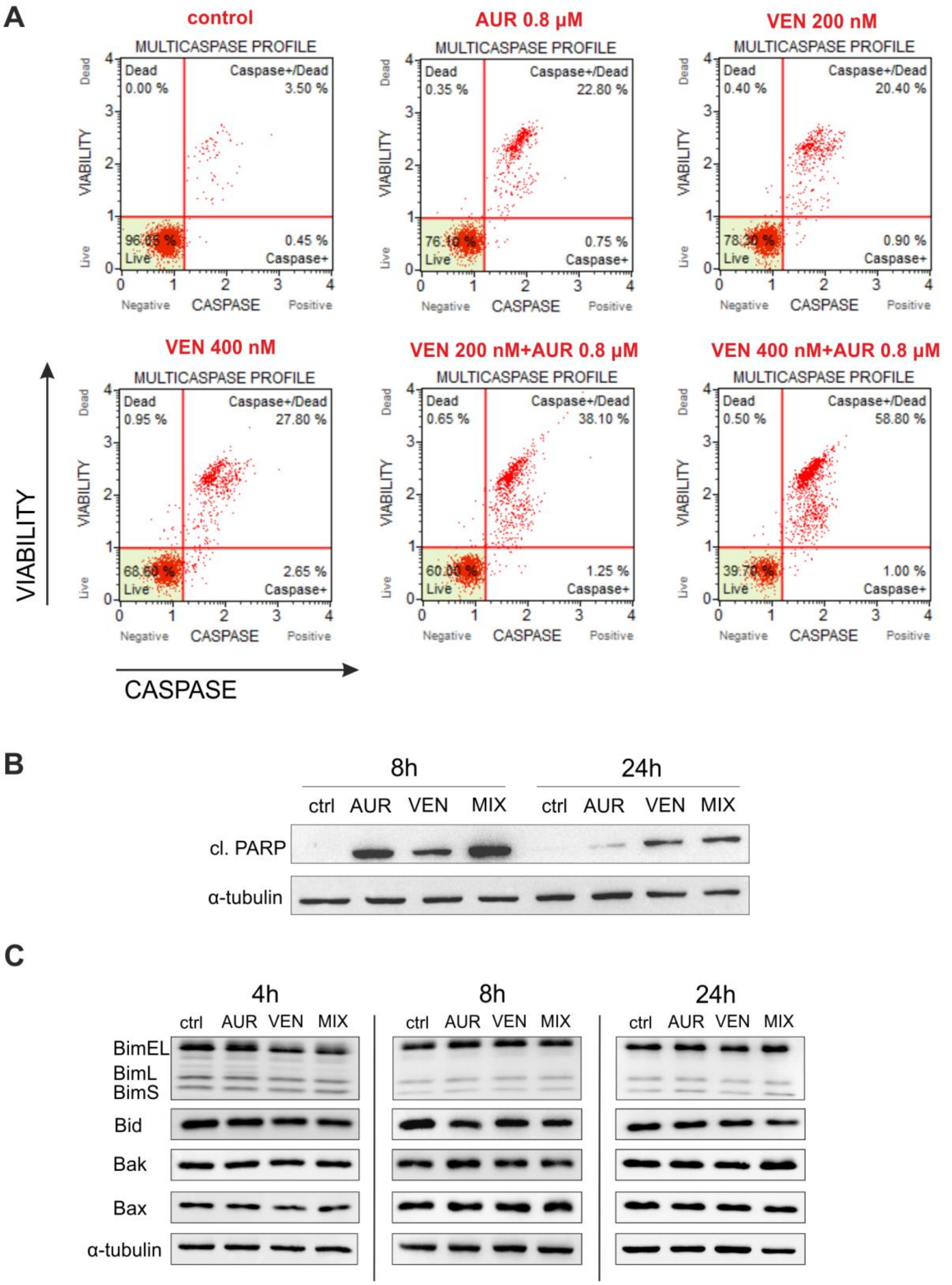

Supplementary Fig. 8. AUR enhances VEN-induced apoptosis. A, SEM cells were treated with VEN, AUR, and both drugs for 16h and subjected to MultiCaspase assay. Activation of caspases and cell death was determined by staining of the cells with a fluorescently-labeled inhibitor of caspases (FLICA) and 7AAD dye and analyzed using Muse Cell Analyzer. Plots present percentages of viable and dead cells with activated caspases. **B,** SEM cells were treated with 0.8 μM AUR, 400 nM VEN, or a combination of both drugs for 8 h and 24 h. The level of cleaved PARP protein in response to the drugs was determined by immunoblotting. An equal sample loading was assessed by detection of

α-tubulin, n=3. **C,** SEM cells were exposed to 0.8 μM AUR, 400 nM VEN, and a combination of both drugs for 4, 8, and 24 h. The levels of members of selected pro-apoptotic BCL-2 family proteins were determined by immunoblotting. Representative blots of at least 2 independent experiments are presented.

Sup. Fig. 9

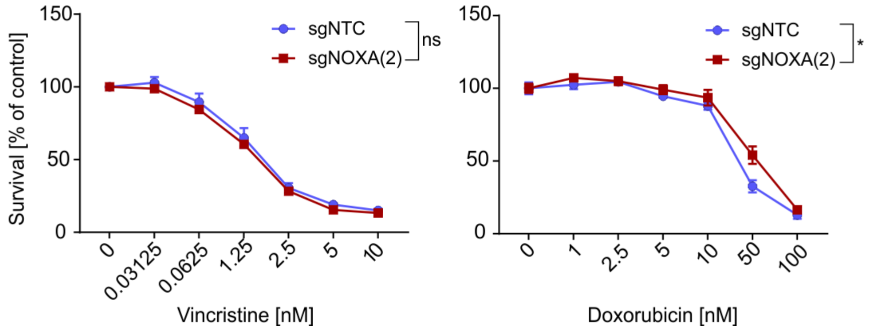

Supplementary Fig. 9. Levels of NOXA protein do not significantly affect sensitivity to vincristine and doxorubicin. SEM cells with *PMAIP1* KO [sgNOXA(2)] and control cells (sgNTC) were exposed to chemotherapeutics, vincristine or doxorubicin, at indicated concentration range for 48 h. The viability of the cells was evaluated by MTT assay. The graphs show the mean percentage of the controls (treated with DMSO) ± SEM, from 8 biological replicates (n=8). *P < 0.05 for the interaction by 2-way ANOVA.

Sup. Fig. 10

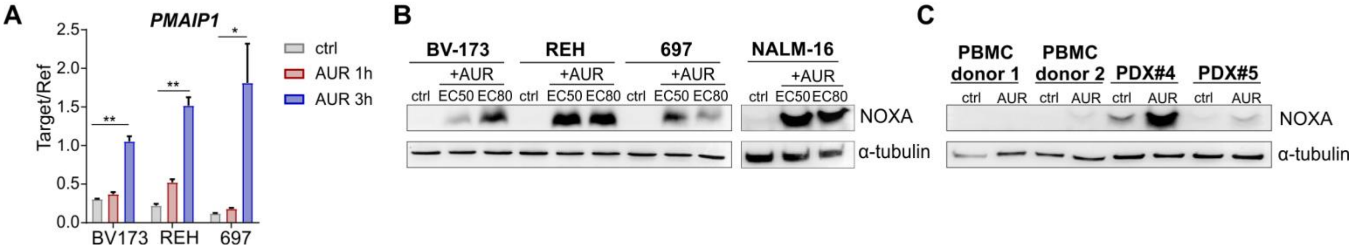

Supplementary Fig. 10. AUR strongly induces NOXA at mRNA and protein levels in various BCP-ALL cell lines. A, The levels of *PMAIP1* mRNA in BCP-ALL cell lines (BV-173, REH, 697) exposed to EC50 concentration of AUR for 1 and 3 h were assessed by RT-qPCR. Data are presented as a mean expression of the target gene relative to *RPL29* and *GUSB* control genes. Bars show mean values + SEM, n=2. *P < 0.05, **P < 0.01 by 1-way ANOVA with Dunnett's post-hoc test. **B,** Same BCP-ALL cell lines as in (A) plus NALM-16 were treated with EC50 or EC80 of AUR and collected after 8 h. NOXA protein levels were assessed by immunoblotting. Representative blots of 2 independent experiments are shown. **C,** Human primary PBMC, PDX#4, and PDX#5 cells were incubated with 1.5 µM AUR and collected after 4 h. NOXA protein levels were assessed by immunoblotting.

Sup. Fig. 11

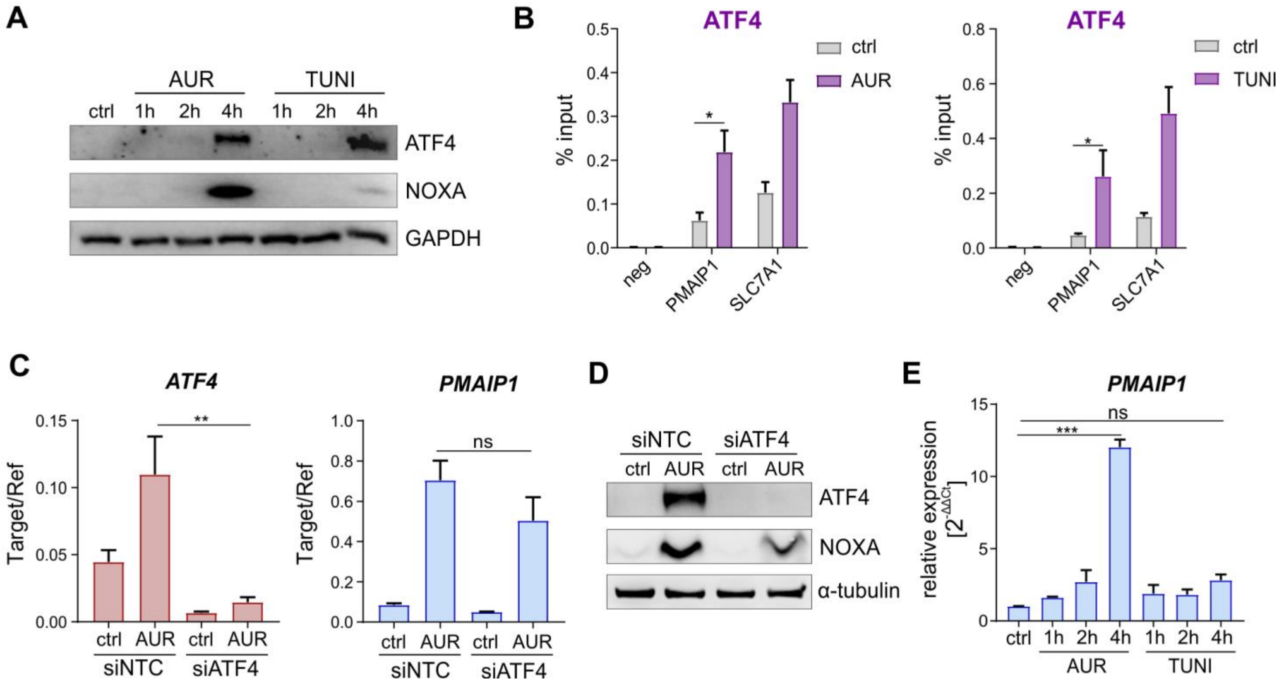

Supplementary Fig. 11. ATF4 only partially contributes to AUR-mediated NOXA upregulation. A, SEM cells were exposed to either 1.5 μM AUR or 5 μg/ml tunicamycin (TUNI) for the indicated period of time. ATF4 and NOXA proteins levels were determined by immunoblotting. GAPDH served as a loading control. **B,** SEM cells were treated with 1.5 μM AUR, 5 μg/ml TUNI or DMSO (control) for 4 h, and ATF4 transcription factor binding within *PMAIP1* loci was determined by ChIP-qPCR. *SCL7A1* is a known ATF4 target and served as a positive control. The bars indicate mean + SEM. *P < 0.05 by paired t-test, n=4. **C, D,** 24 h following siRNA-mediated ATF4 downregulation, SEM cells were exposed to 1.5 μM AUR for 4 h. *ATF4* and *PMAIP1* mRNA expression (**C**), as well as their protein levels (D), were determined in control (siNTC) and in ATF4 siRNA-treated SEM cells (siATF4). The bars indicate the mean relative expression of the target to reference genes (*RPL29*, *GUSB*) + SEM. **P < 0.01 by paired t-test, n=3. **E,** SEM cells were exposed to 1.5 μM AUR or 5 μg/ml tunicamycin (TUNI) for 1, 2, and 4 h. The graph shows mean *PMAIP1* gene expression relative to *GAPDH* over control + SEM, n=2. Mean expression values were calculated using cycle threshold (Ct) values and presented as $2^{-\Delta\Delta Ct}$. ***P < 0.001 by 1-way ANOVA with Dunnett's post-hoc test; ns – not significant.

Supplementary Materials and Methods

Cell culture

Human BCP-ALL cell lines representing various genetic subtypes of the disease were purchased from DSMZ (SEM, RS4;11, BV-173, SUP-B15, SD-1, MUTZ-5, NALM-16) and from ATCC (697, REH), and include hypodiploid cells (NALM-16) as well as cells with specific genetic translocations, such as *MLL-AF4* (SEM, RS4;11), *BCR-ABL1* (BV-173, SUP-B15), *E2A-PBX1* (697), *TEL-AML1* (REH). MUTZ-5 represents a Philadelphia-like subtype carrying *IGH-CRLF2* translocation and a JAK2 R683G mutation. All cell lines were maintained in RPMI 1640 medium (Gibco, Paisley, UK) supplemented with 10% FBS (HyClone, Logan, UT, USA) and 1% penicillin/streptomycin solution (Sigma-Aldrich, St. Louis, MO, USA). Acute myeloid leukemia cell line, HL-60, was purchased from DSMZ and cultured in IMDM medium (Gibco) with 10% FBS and 1% penicillin/streptomycin solution (Sigma-Aldrich). *TP53* deletion in HL-60 was confirmed as described in (1). GFP-expressing OP9 murine bone marrow stromal cell line was kindly provided by Prof. Meinrad Busslinger from The Research Institute of Molecular Pathology (IMP), Vienna, Austria. OP9-GFP cells were cultured in α-MEM medium (Gibco) with 20% FBS (Gibco) and 1% penicillin/streptomycin solution (Sigma-Aldrich). All cell lines were maintained for the maximum time of 4 weeks at 37°C and 5% CO_2 conditions and tested for Mycoplasma contamination once a week.

Reagents

Venetoclax (DC Chemicals, Shanghai, China), auranofin (Sigma-Aldrich), nutlin-3 (Sigma-Aldrich), and tunicamycin (MP Biomedicals, Santa Ana, CA, USA), were dissolved in DMSO. Drug aliquots were stored at -20°C or -80°C. In all experimental procedures control groups were treated with DMSO (Sigma-Aldrich).

MTT assay

The cytostatic/cytotoxic effects of single drugs and drugs combinations in BCP-ALL cell lines were tested using 3-(4,5-dimethylthiazol-2-yl)-2,5-diphenyltetrazolium bromide (MTT) assay. The assay was performed as described in (2) and the cells were exposed to particular drugs for 48 h. Based on the results from single drug treatment (at least 2 independent experiments), EC20 and EC50 values were determined by nonlinear regression dose–response analysis using GraphPad Prism 7. EC20 and EC50 values of each drug were further used for combinatorial drug testing.

Combination index (CI) calculation

In order to determine the potency of drug combinations, combination index (CI) was calculated using CompuSyn software. The software employs Chou-Talalay method as described in (3). The algorithm

defines synergistic (CI < 1), additive (CI = 1) and antagonistic (CI > 1) effects of drug combinations.

Animal studies

All animal procedures were in line with EU Directive 2010/63/EU and the Polish legislation for animal experiments of the Polish Ministry of Science and Higher Education (February 26, 2015) and with ARRIVE guidelines. For animal experiments the sample size was calculated by power analysis or resource equation approach as described in (4). The exclusion criteria were pre-established and aligned with ARRIVE guidelines. The experiments were carried out in SPF animal facility with IVC systems.

Primografts generation

For propagation of primografts, also referred as patient-derived xenografts (PDXs), primary material isolated from BCP-ALL patients was used. The blasts were isolated as described in (2) following the patients' written consent and approval of Bioethics Committee of Medical University of Warsaw and the Medical University of Lodz (KB/44/2015, RNN/51/19/KE). 6-12 weeks old severely immunodeficient NOD.Cg-Prkdc^{scid} Il2rg^{tm1Wjl}/SzJ (NSG) mice (female and male) (Charles River Laboratories, Wilmington, MA, USA) were used for the propagation of BCP-ALL PDXs. All performed animal procedures were approved by the Ethics Committee of the Warsaw University and the Warsaw University of Life Sciences (287/2016, WAW2/095/2019). The detailed experimental procedure and monitoring of PDXs engraftment were previously described (2). See Sup Table 1 and Sup Table 2 for detailed information about generated PDX samples and for selected antibodies.

Drug testing in BCP-ALL PDXs in monoculture

BCP-ALL PDXs were thawed and their viability was determined using trypan blue exclusion method. When the percentage of dead cells exceeded 20%, the viable cell population was enriched using Lymphoprep™ density gradient medium (STEMCELL Technologies, Vancouver, BC, Canada). For that purpose, cells were centrifuged at 550 × g for 25 min and their viability determined once again in a hemocytometer. Successfully enriched cell population was seeded onto 96-well plate, 1.0×10^5 cells per well in 100 µl of SFEM II medium (STEMCELL Technologies) supplemented with 20% of FBS (Gibco), 20 ng/mL of recombinant IL-3 (R&D Systems, Minneapolis, MN, USA), and 10 ng/mL of recombinant IL-7 (R&D Systems). The same day VEN was added to each well at the concentration range between 1 nM – 200 nM in a total volume of 200 µl in 2 repeats. Cells were exposed to the drug for 3 days. In order to determine the absolute number of viable cells after treatment, the cells were stained with human anti-CD19 antibody (BD Biosciences, Franklin Lakes, NJ, USA), and 7AAD (BD Biosciences) following the addition of CountBright™ Absolute Counting Beads (ThermoFisher

Scientific, Waltham, MA, USA) and analysis on CANTO II flow cytometer (BD Biosciences). The number of $CD19^+$ $7AAD^-$ cells was calculated based on the equation provided by the beads' manufacturer. Finally, for each PDX EC50 value of VEN was calculated as described above for BCP-ALL cell lines.

Primary BM-MSC isolation and culture conditions

Before sample collection appropriate individual written consent was obtained and all the procedures were approved by Bioethical Committee of Medical University of Warsaw. The cells were isolated and maintained in culture for the maximum of 5 passages as described in (5). Prior to use in *ex vivo* experiments BM-MSC cells were characterized and tested for the expression of particular positive (CD73, CD105, CD90) and negative (CD45, CD34, CD19) surface markers by flow cytometry and their differentiation capacity as presented in our previous work (2).

Drug testing in BCP-ALL and BM-MSC stromal cells co-culture model

To assess cytotoxic/cytostatic effects of the drugs on MLLr BCP-ALL PDXs in the presence of stromal cells, we employed the co-culture model described in (2, 5), with some modifications. Specifically, bone marrow-derived mesenchymal stem cells (BM-MSC) were seeded onto 96-well plate at 0.2×10^4 cells per well. The next day, after removal of the medium, PDX cells processed as described above for monoculture conditions were seeded onto BM-MSC (1.0×10^5 cells per well in 100 µl of their medium). Next, single drugs or VEN+AUR combination were added (total volume of 200 µl), in 2 independent wells. After additional 4-5 days, the total amount of viable cells was assessed by flow cytometry using CountBright™ Absolute Counting Beads (ThermoFisher Scientific, Waltham, MA, USA). The results are presented as the percentage of untreated control (DMSO). To assess drug interaction, combination index (CI) was calculated using CompuSyn software.

Drug testing in BCP-ALL-OP9 cells co-culture model

The activity of single drugs and VEN+AUR combination on Philadelphia $(Ph)^+$ and Ph-like BCP-ALL cells was tested in the presence of OP9 stromal cells. For this purpose, GFP-expressing OP9 cells were seeded at the density of 0.3×10^4 cell per well onto 96-well plate in 100 µl of their medium. After 6 h leukemic cells were thawed and seeded as depicted for PDXs grown in monoculture. The same day cells were exposed to single and VEN+AUR drugs combination and following 3 days of the treatment they were stained with 7AAD dye. The viable PDX cells were determined by flow cytometry using CANTO II (BD Biosciences) and presented as the percentage of $GFP^-/7ADD^-$ cells. The type and potency of drug interactions was presented as CI, which was calculated as described above.

CD19$^+$ cells isolation and drug testing

Peripheral blood of healthy donors was obtained from Regional Blood and Hemotherapy Center in Warsaw. Normal peripheral blood mononuclear cells (PBMC) were isolated from the blood using density gradient medium – Lymphoprep™ (1.077 g/ml, STEMCELL Technologies). Next, normal CD19$^+$ cells were isolated from PBMC using EasySep™ Human CD19 Positive Selection Kit II (STEMCELL Technologies). For the viability assay, the enriched cells (more than 75% of CD19$^+$ detected by flow cytometry) were seeded onto 96-well plate, 2.0 × 10^5 cells per well in 100 µl of SFEM II medium containing 20% FBS and 1% penicillin/streptomycin. The same day, the cells were treated with VEN, AUR or both (final volume per well - 200 µl) and following 48 h the viable cells were detected by flow cytometry (Fortessa X-20, BD Biosciences) using propidium iodide (PI) dye (final concentration 1 µg/ml, Sigma-Aldrich). The viable cells are presented as the percentage of PI-negative cells, normalized to DMSO-treated control cells.

DNA and RNA isolation

Genomic DNA was extracted from leukemic cells using the Sherlock AX (A&A Biotechnology, Gdansk, Poland). For RT-qPCR analysis of *TP53*, *MDM2* and *CDKN2A* RNA was extracted from BCP-ALL cell lines and PDX cells using RNeasy Mini Kit (QIAGEN, Hilden, Germany) with an additional step of DNase digestion with an RNase-Free DNase Set (QIAGEN), according to manufacturer's protocol. The concentration and quality of isolates were determined by ultraviolet spectrophotometry (NanoDrop 8000, Thermo Scientific, Waltham, MA, USA). RNA integrity (RIN value) was measured by 2200 TapeStation System (Agilent Technologies, Waldbronn, Germany).

Panel-based and Sanger DNA sequencing

Targeted NGS sequencing of leukemic genome was performed using custom designed SureSelect XT-HS2 DNA System panel (Agilent Technologies) comprising 217 genes involved in the pathogenesis of pediatric malignancies. 10 ng DNA was processed according to the manufacturers' protocol. The DNA libraries were prepared according to manufacturer's protocols and sequenced on a NextSeq 550 system (Illumina, San Diego, CA, USA) in the process of 300 bp paired-end run. The raw data analyses were performed using SureCall v. 4.1 software and mapped to the GRCh37/hg19 reference sequence. The presence of the *TP53* mutation in SEM cell line was confirmed using direct Sanger sequencing with the following primers:

For_exon7: 5'-TCCATCTCAAAAAAAAAAAAAAAAAGG-3'

Rev_exon7: 5'-CCCAAAGCCAGAGAAAAGAAAAC-3'.

The analysis of chromatograms was conducted using Sequencher DNA Sequence Analysis Software v. 5.2.0 (GeneCodes Corporation, Ann Arbor, MI, USA).

RT-qPCR

Cell lines:

For RT-qPCR analysis (*PMAIP1, TXNRD1, TXNRD2, ATF4*) RNA was extracted using RNeasy Mini Kit (QIAGEN). In order to remove genomic DNA contamination, 0.5 µg RNA was incubated with DNase (Sigma-Aldrich) and then reverse transcribed with either Transcriptor First Strand cDNA Synthesis Kit (Roche, Mannheim, Germany) or with Superscript III (ThermoFisher Scientific) and random hexamer primers (ThermoFisher Scientific). mRNA levels of *PMAIP1, TXNRD1, TXNRD2, ATF4* and housekeeping genes, *RPL29*, *GUSB or GAPDH* were assessed using LightCycler® 480 SYBR Green I Master (Roche) or Fast SYBR™ Green Master Mix (ThermoFisher Scientific). The relative gene expressions were analysed using LightCycler 480 Software 1.5 (Roche) as described in (6) or calculated based on the cycle threshold (Ct) values as detected in QuantStudio 3 Real-Time PCR System (ThermoFisher Scientific). Primer sequences are listed in Sup Table 3.

Primary samples:

1 µg RNA was used for cDNA synthesis using the High-Capacity cDNA Reverse Transcription kit (ThermoFisher Scientific) and 60 ng cDNA was used for each RT-qPCR reaction. The expression of *TP53*, *MDM2* and *CDKN2A* target genes and *ACTB* and *B2M* as internal control genes was measured in duplicate by a fluorescence-based kinetic RT-qPCR using the following TaqMan™Gene Expression Assays: Hs01034249_m1, Hs00923894_m1, Hs01066930_m1, Hs01060665_g1, Hs00187842_m1 (Applied Biosystems, Foster City, CA, USA) and TaqMan™ Gene Expression Master Mix (Applied Biosystems). The reaction was performed using AriaMx Real-time PCR System (Agilent Technologies) in accordance with the manufacturer’s instructions and data processing was performed using Agilent Aria1.71 software. For the analysis of RT-qPCR data, the relative gene expression (FC – fold change) was calculated using cycle threshold (Ct) values and following the formula: $FC = 2^{-\Delta Ct}$; $\Delta Ct = Ct(target) - Ct(ref)$.

Downregulation of selected genes with siRNA in MLLr BCP-ALL cell lines

SEM or RS4;11 cells were washed twice in Opti-MEM medium (ThermoFisher Scientific), and then 1.0×10^7 cells were subjected to electroporation in Gene Pulser Xcell Electroporation System (Bio-Rad Laboratories, Hercules, CA, USA), under following conditions: 330V, 10ms, 1 pulse, program Square Wave. The electroporation was performed in 400 µl of Opti-MEM medium in Bio-Rad Cuvettes with

0.4 cm gap. For all knockdown experiments ON-TARGETplus Human siRNA, SMARTpool (Dhamacon, Lafayette, CO, USA) were used. The sequences of SMARTpool siRNAs are presented in Sup Table 4. Following electroporation cells were cultured in their standard medium (RPMI + 10% FBS + antibiotics) at the density of 1.0 × 10^6 cells/ml, until further experimental procedures. The viability of electroporated cells exceeded 90% in all knockdown experiments.

Lentiviral modifications of SEM cell line

For the genomic knockout of *PMAIP1* (encoding NOXA), specific sgRNA sequences were designed and cloned into pLentiCRISPRv2 plasmid (Addgene plasmid #52961). These sgRNA sequences are as follows: **sgNOXA(1):** TCGAGTGTGCTACTCAACTC**,** **sgNOXA(2):** ACGCTCAACCGAGCCCCGCG**,** **sgNOXA(3):** TTCTTGCGCGCCTTCTTCCC. The construct encoding sgRNA against the non-mammalian targeting control (NTC, sequence: ACGGAGGCTAAGCGTCGCAA) served as a control. Transfection of human HEK293T cells and lentivirus production was performed as described in (7), however in this study plasmid delivery was performed using polyethylenimine (31.5 µg PEI per 10.5 µg DNA). Supernatant containing lentiviral particles was added to 0.5 × 10^6 of SEM cells and then cells underwent spinoculation for 1 h (750 × g at 32°C) in the presence of 5 µg/ml polybrene. Following transduction, clonal lines were selected using 2 µg/ml puromycin.

ROS detection

Cytosolic ROS levels were assessed using CM-H2-DCFDA dye (Molecular Probes, Eugene, OR, USA) as described in (2) and mitochondrial ROS using MitoSOX Red dye (Molecular Probes). For MitoSOX assay cells were seeded at the density of 0.2 × 10^6 cells per 1 ml and stained with 5 µM of the dye for 10 min at 37°C. In both assays the cells were treated with indicated concentrations of single drugs and VEN+AUR combination for 1, 2 and 4 h.

Caspase activity

0.2 × 10^6 of SEM cells were seeded onto 24-well plate in their medium and treated with AUR, VEN and combination of both for 16 h. The activity of particular caspases (-1, -3, -4, -5, -6, -7, -8, -9) was performed using Muse MultiCaspase Assay Kit (Merck Millipore, Burlington, MA, USA) following the manufacturer's instructions and analysed in Muse Cell Analyzer (Merck Millipore).

Immunoblotting

Whole cell extracts from BCP-ALL cell lines were prepared in Triton X-100-based buffer and subjected to immunoblotting as previously described in (2). Extraction and detection of histone proteins was performed as in (8). Antibodies used for detection of particular proteins are listed in Sup Table 2.

Detection of dead cells by propidium iodide staining

Genetically modified SEM and RS4;11 cells were seeded onto 96-well plates (0.2×10^6 cells/ml density) in their medium and treated with VEN, AUR or combination of both for 24 or 48 h. The cells were then stained with propidium iodide (PI, final concentration of 1 µg/ml) (Sigma-Aldrich) and analysed in Canto II or Fortessa X-20 flow cytometer (BD Biosciences).

ChIP-qPCR

ChIP assay was performed as described in (8, 9). SEM cells were exposed to 1.5 µM AUR for 4 h and 1.0×10^7 cells were fixed [1% formaldehyde for 10 min (histone ChIP), or 2 mM disuccinimidyl glutarate for 30 min followed by 1% formaldehyde for 30 min (ATF4 ChIP)] and then DNA was fragmented on Covaris ME220 sonicator according to the manufacturer's protocol. Next, magnetic isolation of antibody-chromatin complexes (for antibodies used see Sup Table 2) was performed using Protein A and G Dynabeads (ThermoFisher Scientific) following a wash step with a solution of 50 mM HEPES-KOH, pH 7.6, 500 mM LiCl, 1 mM EDTA, 1% NP-40 and 0.7% Na-deoxycholate, which was repeated three times. Finally, the beads were washed once with Tris-EDTA, eluted, and incubated with RNase A and Proteinase K and crosslinks were reversed at 65°C overnight. DNA was then isolated using PCR Purification Kit (QIAGEN) and analysed by qPCR (primer sequences are listed in Sup Table 5). The enriched genomic DNA was normalized to inputs as described in (10). SEM H3K27ac ChIP-seq is taken from GSE74812.

ATAC-seq

SEM cells were exposed to AUR or DMSO for 4 h and then 5×10^4 cells were harvested. For ATAC-seq the samples were processed as described in (8) using the Nextera Tn5 transposase (Illumina). Paired-end sequencing was performed on DNA libraries using NextSeq 500 (Illumina). Quality control of FASTQ reads, alignments, PCR duplicate filtering, blacklisted region filtering and UCSC data hub generation were performed using the NGseqBasic pipeline (11).

RNA-seq

Male and female NOD.Cg-Prkdc^{scid} Il2rg^{tm1Wjl}/SzJ (NSG) mice (Charles River Laboratories) were injected intravenously with 0.5×10^6 PDX#1 cells (derived from adult MLL-AF4+ BCP-ALL patient). Leukemia engraftment was detected once a week and determined as percentage of human blasts in murine peripheral blood using specific antibodies and flow cytometry. Once the percentage of human CD45$^+$/CD19$^+$ cells among murine CD45$^+$ cells exceeded 1% (26 days post engraftment), the mice were randomly selected and treated with either control (60% Phosal 50PG; 30% PEG400;

10% ethanol), or 100mg/kg of VEN (oral gavage). The drugs were given 5 times per week for a total of 13 doses and the mice were sacrificed at the last day of the treatment. The spleens were harvested from all mice and leukemic cells were isolated using EasySep™ Human CD19 Positive Selection Kit II (STEMCELL Technologies). After the magnetic separation, the total amount of viable $CD19^+$ cells was evaluated in trypan blue (>90% viable cells). The cells were washed in PBS and dissolved in Trizol for RNA isolation. RNA was isolated from leukemic cells according to manufacturer’s protocol (Ambion, Life Technologies, Carlsbad, CA) with an additional step of DNase digestion with an RNase-Free DNase Set (QIAGEN). The concentration and quality of isolates were determined by ultraviolet spectrophotometry (NanoDrop 8000, ThermoFisher Scientific). RNA integrity (RIN value) was measured by 2200 TapeStation System (Agilent Technologies).

RNA sequencing was performed using TruSeq Stranded mRNA (Illumina). 1 µg RNA was processed according to the manufacturers' protocol. Briefly, polyA containing mRNA molecules were purified using oligo-dT attached magnetic beads in two rounds of purification. Then, RNA was fragmented and primed for cDNA synthesis followed by the second strand synthesis resulting in blunt-ended cDNA. Secondly, adapter ligation to the ends of the ds cDNA fragments was performed and samples were amplified with PPC (PCR Primer Cocktail) that anneals to the ends of the adapters, and cleaned-up. In order to optimize cluster densities across every lane of the flow cell, quantification of DNA libraries using DNA 1000 chip on an Agilent Technologies 2200 TapeStation System was performed. Indexed DNA libraries were normalized to 4 nM and then pooled in equal volumes in the PDP plate. Library was sequenced on a NextSeq 550 system (Illumina) using NextSeq® Reagent Kit v3 (150 cycles) with a TG NextSeq 500/550 High Output Flow Cell. Images from the instrument were processed to generate FASTQ sequence files.

FASTQ files were quality checked using FastQC (v0.11.4). Adapters and poor-quality bases were then trimmed from the reads using trim_galore (v0.4.1). Paired-end reads were mapped to the human genome assembly (hg19) using the STAR aligner (v2.4.2). PCR duplicates were removed using picard-tools MarkDuplicates (v1.83). Mapped reads were then quantified over gene exons using subread featureCounts (v1.6.2) to measure gene expression levels.

Statistical analysis was performed in R using the edgeR package (12), and P values were corrected using the Benjamini & Hochberg method (13) to acquire False Discovery Rates (FDR). Genes were considered differentially expressed if they had an FDR of less than 0.05.

Differentially expressed genes were analysed for enriched pathways using PANTHER (v15) (14). p53 pathway genes for visualisation/exploration were extracted from the PANTHER database (14), and the KEGG database (hsa04115). Data visualisations were performed in R. Heatmaps were

visualised using $\log_2$ CPM expression values that were Z-score transformed, such that the data are represented by the standard deviations from mean expression.

VEN-treatment *in vivo* and RT-qPCR

Male and female NOD.Cg-Prkdc^{scid} Il2rg^{tm1Wjl}/SzJ (NSG) mice (Charles River Laboratories) were injected intravenously with MLLr BCP-ALL PDX samples as follows: PDX#2 (0.5×10^6 cells/mouse), PDX#3 (5.0×10^6 cells/mouse), PDX#4 (5.0×10^6 cells/mouse), PDX#5 (5.0×10^6 cells/mouse), and PDX#6 (2.0×10^6 cells/mouse). Leukemia engraftment was detected by flow cytometry using specific antibodies as described above. When the percentage of human blasts in the murine blood reached around 1%, the mice were randomly selected and treated with either vehicle control (60% Phosal 50PG; 30% PEG400; 10% ethanol), or 100mg/kg of VEN (oral gavage). The mice received the drug for a total of 11 doses (PDX#2), 12 doses (PDX#3, PDX#4), 15 doses (PDX#6), or 20 doses (PDX#5) and were sacrificed at the last day of the treatment. Next, human CD19$^+$ leukemic cells were isolated from the spleens of control and VEN-treated mice with EasySep™ Human CD19 Positive Selection Kit II (STEMCELL Technologies). These include mice with limited VEN efficacy (from PDX#2 and PDX#3). PBS-washed cell pellets were then dissolved in Trizol for RNA isolation. RNA isolation and quality check steps were performed as described in "RNA-seq" methods section. The expressions of selected p53 pathway-related genes were assessed by RT-qPCR using gene-specific TaqMan probes (TaqMan™Gene Expression Assays, Applied Biosystems) (Sup Table 3). The reaction was performed using AriaMx Real-time PCR System (Agilent Technologies) in accordance with the manufacturer's instructions and data processing was performed using Agilent Aria1.71 software. For the analysis of RT-qPCR data, the relative gene expression (FC – fold change) was calculated using cycle threshold (Ct) values and following the formula: $FC = 2^{-\Delta}Ct$; $\Delta Ct = Ct(target) - Ct(ref)$. RT-qPCR analysis of genes involved in p53 pathway was performed on three PDXs including the one previously subjected to RNA-seq analysis (PDX#1).

Data availability

RNA-seq and ATAC-seq data have been deposited in the Gene Expression Omnibus (GEO) under accession number GSE163229.

Statistical Analysis

The n numbers provided in all figure legends state the number of independent biological replicates (cell lines) or the number of individual samples (patients and animal data). Statistical analyses were performed using GraphPad Prism 7 Software (La Jolla, CA, USA), and p values were considered

statistically significant when lower than 0.05.

The type of statistical test used is described in Figure legends. All tests were two-sided and for multiple comparisons appropriate corrections were adjusted, as indicated in Figure legends. The exclusion criteria were pre-established. Samples/experiments exclusion from a given analysis was performed only when positive or negative controls did not give appropriate results.

References:

1. Deregowska A, Pepek M, Pruszczyk K, Machnicki MM, Wnuk M, Stoklosa T. Differential Regulation of Telomeric Complex by BCR-ABL1 Kinase in Human Cellular Models of Chronic Myeloid Leukemia—From Single Cell Analysis to Next-Generation Sequencing. Genes (Basel). 2020; 11(10):1145.

2. Fidyt K, Pastorczak A, Goral A, Szczygiel K, Fendler W, Muchowicz A, et al. Targeting the thioredoxin system as a novel strategy against B-cell acute lymphoblastic leukemia. Mol Oncol. 2019; 13(5):1180–95.

3. Chou T. Drug Combination Studies and Their Synergy Quantification Using the Chou-Talalay Method. Cancer Res. 2010; 70(2):440–6.

4. Charan J, Kantharia ND. How to calculate sample size in animal studies? J Pharmacol Pharmacother. 2013; 4(4):303–6.

5. Pal D, Blair HJ, Elder A, Dormon K, Rennie KJ, Coleman DJL, et al. Long-term in vitro maintenance of clonal abundance and leukaemia-initiating potential in acute lymphoblastic leukaemia. Leukemia. 2016; 30(8):1691–700.

6. Muchowicz A, Firczuk M, Wachowska M, Kujawa M, Jankowska-Steifer E, Gabrysiak M, et al. SK053 triggers tumor cells apoptosis by oxidative stress-mediated endoplasmic reticulum stress. Biochem Pharmacol. 2015; 93(4):418–27.

7. Bajor M, Zych AO, Graczyk-Jarzynka A, Muchowicz A, Firczuk M, Trzeciak L, et al. Targeting peroxiredoxin 1 impairs growth of breast cancer cells and potently sensitises these cells to prooxidant agents. Br J Cancer. 2018; 119(7):873–84.

8. Godfrey L, Crump NT, Thorne R, Lau IJ, Repapi E, Dimou D, et al. DOT1L inhibition reveals a distinct subset of enhancers dependent on H3K79 methylation. Nat Commun. 2019; 10(1):1–15.

9. Benito JM, Godfrey L, Kojima K, Hogdal L, Wunderlich M, Geng H, et al. MLL-Rearranged Acute Lymphoblastic Leukemias Activate BCL-2 through H3K79 Methylation and Are Sensitive to the BCL-2-Specific Antagonist ABT-199. Cell Rep. 2015; 13(12):2715–27.

10. Milne TA, Zhao K, Hess JL. Chromatin Immunoprecipitation (ChIP) for Analysis of Histone Modifications and Chromatin-Associated Proteins. In: Eric So CW, editor. Leukemia: Methods and Protocols. Totowa, NJ: Humana Press; 2009. p. 409–23.

11. Telenius J, Hughes JR. NGseqBasic - a single-command UNIX tool for ATAC-seq, DNaseI-seq, Cut-and-Run, and ChIP-seq data mapping, high-resolution visualisation, and quality control. bioRxiv. 2018; 393413. Available from: http://biorxiv.org/content/early/2018/08/16/393413.abstract

12. Robinson MD, McCarthy DJ, Smyth GK. edgeR: a Bioconductor package for differential expression analysis of digital gene expression data. Bioinformatics. 2010; 26(1):139–40.

13. Benjamini Y, Hochberg Y. Controlling the False Discovery Rate: A Practical and Powerful Approach to Multiple Testing. J R Stat Soc Ser B. 1995; 57(1):289–300.

14. Thomas PD, Campbell MJ, Kejariwal A, Mi H, Karlak B, Daverman R, et al. PANTHER: a library of protein families and subfamilies indexed by function. Genome Res. 2003; 13(9):2129–41.

Supplementary Tables

Supplementary Table 1. Primary (ALL) and primograft (PDX) BCP-ALL

**Sample no**	**BCP-ALL subtype**	**Risk group** (HR- high risk, ST – standard risk)	**Age at diagnosis [years]**	**Sex**	**Dignosis – D Relapse – R**
ALL#1	undefined	HR	55	female	D
ALL#2	undefined	HR	20	male	D
ALL#3	ETV6-RUNX1	SR	3	female	D
ALL#4	ETV6-RUNX1	SR	5	male	D
ALL#5	undefined	SR	4	male	D
ALL#6	ETV6-RUNX1	SR	3.5	male	D
PDX#1	MLL-AF4	HR	48	female	D
PDX#2	MLL-ENL	HR	3 months	female	R
PDX#3	MLL-AF4	HR	1.5	female	R
PDX#4	MLL-AF4	HR	4	female	D
PDX#5	MLL-AF4	HR	3.5	male	R
PDX#6	MLL-AF4	HR	1.5 month	male	D
PDX#7	MLL-AF4	HR	11	female	D
PDX#8	MLL-AF4	HR	29	female	D
PDX#9	Ph+	HR	41	male	D
PDX#10	Ph+	HR	69	female	R
PDX#11	Ph-like	HR	9	male	D
PDX#12	Ph-like	HR	14	male	R
PDX#13	Ph-like	HR	11.5	male	R
PDX#14	undefined	SR	72	male	D
PDX#15	undefined	SR	40	female	D

Supplementary Table 2. Antibodies; abbreviations: m - mouse, h - human

Antibody	Clone	Catalog no	Company
**Antibodies for flow cytometry**			
anti-mCD45 APC	30-F11	17-0451-83	eBioscience
anti-hCD45 PE	HI30	12-0459-42	eBioscience
anti-hCD34 APC	4H11	17-0349-42	eBioscience
anti-hCD19 FITC	HIB19	11-0199-42	eBioscience
anti-hCD19 APC	HIB19	555415	BD Biosciences
anti-hCD10 PE	CB-CALLA	12-0106-42	eBioscience
**Antibodies for immunoblotting and/or ChIP-qPCR**			
anti-NOXA	D8L7U	14766	Cell Signaling
anti-MCL1	D35A5	5453	Cell Signaling
anti-BCL2	D55G8	4223	Cell Signaling
anti-BCL-XL	54H6	2764	Cell Signaling
anti-BIM	C34C5	2933	Cell Signaling
anti-BID	polyclonal	2002	Cell Signaling
anti-BAK	D4E4	12105	Cell Signaling
anti-BAX	D2E11	5023	Cell Signaling
anti-TXNRD1	19A1	ab16847	Abcam
anti-TXNRD2	3F2-E12-F10	12029	Cell Signaling
anti-p53	1C12	2524	Cell Signaling
anti-p21 Waf1/Cip1	12D1	2947	Cell Signaling
anti-cleaved PARP	D64E10	5625	Cell Signaling
anti- H2AK119ub	D27C4	8240	Cell Signaling
anti-H3K27ac	polyclonal	C15410196	Diagenode
anti-ATF4	D4B8	11815	Cell Signaling
anti-α-tubulin	DM1A	CP06	Calbiochem
anti-β-actin	AC-15	A3854	Sigma-Aldrich
anti-GAPDH	polyclonal	A300-641A	Bethyl Laboratories

Supplementary Table 3. List of primers and TaqMan probes used for RT-qPCR

Primers (SYBR Green)	Sequences
PMAIP1_F	CAGGAGATTTGGAGACAAAC
PMAIP1_R	CTCATGCAAGTTTTTGATGC
TP53_F	AGTCTACCTCCCGCCATAAA
TP53_R	GGGAACAAGAAGTGGAGAATGT
TXNRD1_F	TCACCCCAGTTGCAATCC
TXNRD1_R	GGTTGGAACATTTTCATAGTCACA
TXNRD2_F	CAGCGGGACTATGATCTCCT
TXNRD2_R	AGGTTCCACGTAGTCCACCA
ATF4_F	CCTAGGTCTCTTAGATGATTACC
ATF4_R	CAAGTCGAACTCCTTCAAATC
RPL_F	CAGCTCAGGCTCCCAAAC
RPL_R	GCACCAGTCCTTCTGTCCTC
GUSB_F	GAAAATATGTGGTTGGAGAGCTCATT
GUSB_R	CGGAGTGAAGATCCCCTTTTTA
GAPDH_F	AACAGCGACACCCATCCTC
GAPDH_R	CATACCAGGAAATGAGCTTGACAA
**TaqMan™Gene Expression Assays**	**Assay ID**
BBC3	Hs00248075_m1
TNFRSF10B	Hs00366272_m1
CDKN1A	Hs00355782_m1
FAS	Hs00236330_m1
ZMAT3	Hs00536976_m1
DDB2	Hs00172068_m1
SESN1	Hs00902787_m1
BAX	Hs00180269_m1
TP53I3	Hs00936520_m1
TNFRSF10D	Hs00388742_m1
SESN2	Hs00230241_m1
CDC25A	Hs00947994_m1
CHEK1	Hs00967506_m1
GTSE1	Hs01126673_m1
ACTB	Hs01060665_g1

Supplementary Table 4. Sequences of ON-TARGETplus Human siRNA, SMARTpool

Gene	5’→ 3’
**NTC**	1) UGGUUUACAUGUCGACUAA<br>2) UGGUUUACAUGUUGUGUGA<br>3) UGGUUUACAUGUUUUCUGA<br>4) UGGUUUACAUGUUUUCCUA
**TXNRD1**	1) GCAUCAAGUUUAUAAGACA<br>2) GCGAUAUAUUGGAGGAUAA<br>3) CUAAGGAGGCAGCCCAAUA<br>4) GGACAGCACAAUUGGAAUC
**TXNRD2**	1) GCAUCCCAGUGUUAUGUAA<br>2) GCGAAGUUACUCAAGGAUU<br>3) AGGAGCAUGUUGAGGUCUA<br>4) GCACACGGUUUGCGGCGUU
**TP53**	1) GAAAUUUGCGUGUGGAGUA<br>2) GUGCAGCUGUGGGUUGAUU<br>3) GCAGUCAGAUCCUAGCGUC<br>4) GGAGAAUAUUUCACCCUUC
**ATF4**	1) CAGAUUGGAUGUUGGAGAA<br>2) CGACUUGGAUGCCCUGUUG<br>3) GAAGAACGAGGCUCUAAAA<br>4) GAGAUAGGAAGCCAGACUA

Supplementary Table 5. Sequences of primers used for ChIP-qPCR

Primers	Sequences
PMAIP1_F (H3K27ac ChIP)	GGCGCCTCCAGAAAGTT
PMAIP1_R (H3K27ac ChIP)	CTGCATCCCAATCGCAAATC
CDK6_F (H3K27ac ChIP)	TCGAAGCGAAGTCCTCAACA
CDK6_R (H3K27ac ChIP)	GCTTGGGCAGAGGCTATGTA
PMAIP1_F (ATF4 ChIP)	GCTGTTGCATCAGACGATTATAC
PMAIP1_R (ATF4 ChIP)	CAACTTAGGCATGGTCACATTT
SLC7A1_F (ATF4 ChIP)	CACGCTTACTCACTCGGTGT
SLC7A1_R (ATF4 ChIP)	GCCTCCACGGAGTCCATTTT
PMAIP1_F (H2AK119ub ChIP)	TTCCTCCTGGTTAAACCCTTC
PMAIP1_R (H2AK119ub ChIP)	GGCTTTGACCATCTGCAAAC
HOXC8_F (H2AK119ub ChIP)	CAGAAGGGTAGATAGGAGCCTGC
HOXC8_R (H2AK119ub ChIP)	TCAAACAGCGAAGGAGAGGAAG
Negative control region (neg_F)	GGCTCCTGTAACCAACCACTACC
Negative control region (neg_R)	CCTCTGGGCTGGCTTCATTC
